# Neuronal Subtypes and Connectivity of the Adult Mouse Paralaminar Amygdala

**DOI:** 10.1523/ENEURO.0119-24.2024

**Published:** 2024-06-12

**Authors:** David Saxon, Pia J. Alderman, Shawn F. Sorrells, Stefano Vicini, Joshua G. Corbin

**Affiliations:** ^1^Center for Neuroscience Research, Children’s Research Institute, Children’s National Hospital, Washington, DC 20011; ^2^Interdisciplinary Program in Neuroscience, Georgetown University Medical Center, Washington, DC 20007; ^3^Department of Neuroscience, University of Pittsburgh, Pittsburgh, Pennsylvania 15260; ^4^Center for the Neural Basis of Cognition, University of Pittsburgh, Pittsburgh, Pennsylvania 15260; ^5^Department of Pharmacology and Physiology, Georgetown University Medical Center, Washington, DC 20007

**Keywords:** adolescence, paralaminar amygdala

## Abstract

The paralaminar nucleus of the amygdala (PL) comprises neurons that exhibit delayed maturation. PL neurons are born during gestation but mature during adolescent ages, differentiating into excitatory neurons. These late-maturing PL neurons contribute to the increase in size and cell number of the amygdala between birth and adulthood. However, the function of the PL upon maturation is unknown, as the region has only recently begun to be characterized in detail. In this study, we investigated key defining features of the adult mouse PL; the intrinsic morpho-electric properties of its neurons, and its input and output circuit connectivity. We identify two subtypes of excitatory neurons in the PL based on unsupervised clustering of electrophysiological properties. These subtypes are defined by differential action potential firing properties and dendritic architecture, suggesting divergent functional roles. We further uncover major axonal inputs to the adult PL from the main olfactory network and basolateral amygdala. We also find that axonal outputs from the PL project reciprocally to these inputs and to diverse targets including the amygdala, frontal cortex, hippocampus, hypothalamus, and brainstem. Thus, the adult mouse PL is centrally placed to play a major role in the integration of olfactory sensory information, to coordinate affective and autonomic behavioral responses to salient odor stimuli.

## Significance Statement

Mammalian amygdala development includes a growth period from childhood to adulthood, believed to support emotional and social learning. This amygdala growth is partly due to the maturation of neurons during adolescence in the paralaminar amygdala (PL). However, the functional properties of these neurons are unknown. In our previous studies, we characterized the paralaminar amygdala in the adolescent mouse. Here, we investigate the properties of the adult PL in the mouse, revealing the existence of two neuronal subtypes that may play distinct functional roles in the adult brain. We further reveal the brain-wide input and output connectivity of the PL, indicating that the PL integrates olfactory cues for emotional processing and delivers information to regions associated with reward and autonomic states.

## Introduction

The paralaminar nucleus (PL) is a subregion of the mammalian amygdala containing neurons that are born prenatally but delay their maturation until adolescence (deCampo and Fudge, 2012; Sorrells et al., 2019; Alderman et al., 2024). The late development of PL neurons contributes to a hallmark increase in human amygdala size and neuron number between childhood and adulthood (Avino et al., 2018). The PL was originally identified in the human (Crosby and Humphrey, 1941) and later in the monkey, sheep, tree shrew, and rat (Nacher et al., 2002; Fudge, 2004; Chareyron et al., 2012; Martí-Mengual et al., 2013; Piumatti et al., 2018; Ai et al., 2021). We recently identified its homolog in the mouse, revealing similarities in its developmental and molecular features across the mouse and human (Alderman et al., 2024).

The mouse PL is located rostrally to the basolateral amygdala and ventral to the striatum. It is distinguished from nearby regions by its dense arrangement of small neurons that express markers of immaturity (Dcx, Psa-Ncam) and excitatory neurons (VGlut1, VGlut2, Tbr1) during juvenile ages [postnatal day (P)7–21]. By early adulthood (P60), mouse PL neurons have matured (Dcx−, NeuN+), and most (∼84%) mature PL neurons express the excitatory marker Tbr1. Thus, across mammalian species, the PL is a nucleus of late-maturing excitatory neurons, likely influencing amygdala function upon maturation. However, beyond histological analyses, the adult PL has not been well studied.

While the circuit connectivity and functional role(s) of late-maturing neurons in the PL are unknown, postnatally immature neurons exist and have been studied in other brain regions (Benedetti et al., 2023). These regions include the rodent dentate gyrus and olfactory bulb, where, in contrast to the PL, the generation of newborn neurons occurs postnatally (Sakamoto et al., 2014; Denoth-Lippuner and Jessberger, 2021), and the non-neurogenic piriform cortex, where prenatally born neurons undergo gradual maturation similar to the PL (Gómez-Climent et al., 2008; Rotheneichner et al., 2018; Ghibaudi et al., 2023). In each of these regions, immature cells develop postnatally into mature neurons with functional synaptic input (Li et al., 2018; Dieni et al., 2019; Benedetti et al., 2020). This integration is widely believed to contribute to activity-dependent modality-specific learning (Benedetti and Couillard-Despres, 2022; Borzello et al., 2023; Chaker et al., 2023). The mouse PL develops a responsiveness to olfactory stimulation between juvenile and adult ages (Alderman et al., 2024), suggesting that PL neurons become functionally integrated during maturation.

The identity of a neuron within a circuit is defined by both intrinsic and extrinsic characteristics. In addition to gene expression profiles, intrinsic characteristics include electrophysiological and morphological properties, which in many brain regions are tied to divergent roles (Hattox and Nelson, 2007; Ascoli et al., 2008; Sun et al., 2013; Gouwens et al., 2019; S. Chen et al., 2022). Extrinsic characteristics, defined by the regions and patterns of input and output connectivity, shape neuronal function within an interconnected circuit (Zeng, 2018) and are of particular interest in dissecting late-maturing populations (Benedetti and Couillard-Despres, 2022; Borzello et al., 2023). While our past studies have investigated the developing intrinsic properties in the PL at juvenile ages (Alderman et al., 2024), the electrophysiological profiles, morphologies, and connections that define the mature PL in adulthood are unknown. Such knowledge is critical to understanding the function of PL neurons in adult behavior.

Using a combination of electrophysiological, morphological, and anatomical tracing approaches, in this study we define the intrinsic neuronal properties of the adult PL as well as its input and output projections. We find that mature PL neurons are not uniform but instead comprise two excitatory neuronal subtypes defined by distinct action potential firing patterns and dendritic branching properties. We further uncover bidirectional connections between the PL and the olfactory network and basolateral amygdala, with additional outputs to regions involved in memory, reward, visceral, and autonomic states. Together, our results detail the diversity of excitatory neurons as well as the major input and output connections that define this uniquely late-developing region of the amygdala.

## Materials and Methods

### Patch-clamp electrophysiology

Adult (P59-P78) C57/Bl6 mice (Jax #000664; *n* = 12 males, 12 females) were anesthetized with isofluorane and decapitated. Brains were dissected and placed into ice-cold cutting ACSF, containing the following (in mM): 234 sucrose, 11 d-glucose 11, 2.5 KCl, 26 NaHCO_3_, 1.25 NaH_2_PO_4_, 7 MgSO_4_, 0.5 CaCl_2_. Sagittal sections (275 µm thick) were sliced using a Leica V1200 Vibratome. Sections containing the PL were transferred to recording ACSF, containing the following (in mM): 125 NaCl, 3.5 KCl, 2 CaCl_2_, 1 MgCl_2_, 1.25 NaH_2_PO_4_, 26 NaHCO_3_, 10 d-glucose 10. This solution was initially held at 34°C for 30 min to facilitate slice recovery, before cooling to room temperature for the duration of the experiment. All solutions were adjusted to pH 7.4 and 300 mOsm and were bubbled with carbogen (95% CO_2_/5% O_2_) during all steps. All chemicals were supplied by Sigma.

Patch pipettes (5–9 MΩ) were pulled from borosilicate glass pipettes (Sutter Instruments) using a vertical two-stage puller (Narishige PP-830) and filled with an internal solution containing the following (in mM): 145 K-gluconate, 10 HEPES, 1 EGTA, 2 Mg-ATP, 0.3 Na_2_-GTP, and 2 MgCl_2_ (Thermo Fisher Scientific). This solution was adjusted to pH 7.3 and 290–300 mOsm, and 0.5% biocytin was added on the day of the experiment.

Patch-clamp electrophysiology was conducted using a Nikon E600 upright microscope and a 60× water immersion objective, and slices were held in a chamber containing recording ACSF with a flow rate of 2–3 ml/min at room temperature. The PL was identified by its anatomical position between the ventral endopiriform cortex, striatum and basolateral amygdala, and its higher cell density than nearby regions.

Whole-cell patch-clamp recordings were obtained using a MultiClamp 700A amplifier, Digidata 1550B digitizer, and pClamp 10 software (Axon Instruments, Molecular Devices). Electrical signals were sampled at 20 kHz. No bridge balance compensation was applied. During whole-cell current-clamp recordings, cells with access resistance >30 MΩ or resting membrane potential drift >5 mV were discarded. One-second-long hyperpolarizing and depolarizing current steps were applied, in increasing intervals of 10 pA up to ±200 pA. Next, voltage-clamp recordings were obtained to record sEPSCs (inward currents at −70 mV holding potential) and sIPSCs (outward currents at −40 mV holding potential). sIPSCs were recorded during periods where no escaped spikes occurred. The following steps were taken to facilitate biocytin filling: each whole-cell configuration was maintained for at least 10 min, after which the pipette was slowly removed from the cell surface over a course of 2–3 min. Slices remained in the chamber for another 15 min before proceeding to fixation (see below, Tissue processing).

### Surgeries

All procedures were conducted in accordance with and approved by the Institutional Animal Care and Use Committee at Children's National Hospital. For all surgeries, C57/Bl6 mice (Jax #000664; P50–80) were anesthetized with isoflurane and placed into a stereotaxic apparatus (Stoelting #51600). Body temperature was maintained with a heating pad during surgery and recovery, and 1–2% isoflurane was delivered continuously through a nose port. Animals were treated with analgesic buprenorphine (0.09 mg/kg body weight of 0.03 mg/ml buprenorphine prepared in sterile saline) prior to surgery and every 12 h afterward as needed. Mice were monitored daily and sacrificed 3–4 weeks post viral injection.

All injection coordinates were measured from bregma. For all injections, the syringe remained in place for 2–4 min following delivery of virus and then slowly withdrawn. For retrograde tracing of PL inputs, AAV2-retro virus (rAAV1-retro-EF1a-DO_DIO_TdTomato_EGFP-WPRE-pA, Addgene #37120, 1 × 10^13^ GC/ml; Tervo et al., 2016) was injected bilaterally (50 nl at 20 nl/min) into the PL (AP: −0.4, ML: ±2.8, DV: −4.75). For anterograde tracing of input sources, rAAV5-hsyn-mCherry-WPRE (Addgene #114472, Titer: 8 × 10^12^ GC/ml) was injected unilaterally (60–100 nl at 40 nl/min) to the following coordinates: BLA (AP: −1.5, ML: ±2.9, DV: −4.7), COA (AP: −2.4, ±2.9, −5.4), nLOT (AP: −0.6, ML: ±1.9, DV: −5.5), ENT (AP: −4.6, ±3.2, −3.6), AIC (AP: 2.2, ML: ±2.1, DV: −2.6). For anterograde tracing of PL outputs, AAV1-phSyn1(S)-FLEX-TdTomato-T2A-Syp-EGFP (Addgene #51509, 4 × 10^14^ GC/ml) was injected (60 nl at 20 nl/min) to adult (P54–112) *VGlut1-ires-cre* mice (Jax #037512).

### Tissue processing

Mice were anesthetized with isoflurane and transcardially perfused with 20 ml of 1× phosphate-buffered saline (PBS) followed by 20 ml of fixative (4% paraformaldehyde in 1× PBS). Following perfusion, brains were kept overnight in fixative at 4°C with shaking and cryoprotected in a solution containing 30% sucrose in PBS for 24–48 h at 4°C with shaking. Next, whole brains were embedded in OCT compound (Fisher Healthcare) and kept at −80°C. Sagittal sections were cut at 50 µm using a cryostat (Leica CM3050S) and stored in PBS containing 0.02% sodium azide.

For immunolabeling, sections were washed in PBS with 0.2% Triton (PBST) and then incubated for 2 h at RT in a blocking solution containing PBST with 5% Normal Donkey Serum. After blocking, primary antibodies were added to fresh blocking solution, and sections were incubated overnight at 4°C. The following primary antibodies were used: Rabbit anti-Tbr1 (1:500, EMD Millipore 10554), goat anti-Tdtomato (1:500, Sicgen AB8181), rat anti-GFP (1:1000, Nacalai-Tesque 0404), guinea pig anti-VGlut2 (1:500, Millipore AB2251). Sections were then washed with PBST, three times for 5 min per wash, and two times for 10 min per wash. Then, sections were incubated in secondary antibodies in fresh blocking solution, for 3 h at room temperature. The following secondaries were used: Donkey anti-chicken Alexa-Fluor 647 (1:1,000, Jackson Immuno 703-605-155), donkey anti-goat Cy3 (1:1,000, Jackson Immuno 705-165-003), donkey anti-rat FITC (1:1,000, Jackson Immuno 712-095-150), donkey anti-guinea pig Alexa-Fluor 488 (1:1,000, Jackson Immuno 706-545-148). Following secondary incubation, sections were again washed with PBST, three times for 5 min per wash, and two times for 10 min per wash. Finally, sections were mounted to slides and coverslipped with Fluoromount-G with DAPI (SouthernBiotech).

For post hoc biocytin labeling of patch-clamp slices, after recordings sections were fixed in 4% PFA overnight at 4°C and then washed in PBST. Fixed sections were incubated overnight at 4°C in fresh PBST containing Streptavidin-Cy5 (1:500, VectorLabs SA-1500-1) and then washed with PBST three times for 10 min per wash and two times for 30 min per wash. Finally, sections were mounted to slides, allowed to dry completely, and coverslipped with Fluoromount-G with DAPI (SouthernBiotech).

### Microscopy

All imaging was performed using a Nikon A1 confocal microscope. Biocytin-filled neurons were imaged using 10× (NA 0.4), 20× (NA 0.75), and 60× (NA 1.4) objectives. *Z*-stack mosaics (*Z*-step 1.05 µm) acquired at 20× were used for morphological reconstruction, and 60× *Z*-stacks (Z-step 0.125 µm) were used for soma reconstruction. For viral-traced tissue, 10× mosaics of whole slides were first acquired with a large pinhole size (45.8 µm), followed by 20× (NA 0.75) and 40× (NA 1.3) *Z*-stacks of regions of interest. Quantification of input neuron sources (Fig. 3) was performed by counting labeled neurons within each region using 10× objective in epifluorescence mode. To visualize VGlut2-expressing synaptic terminals within the PL (Fig. 4), the 60× objective was used with an additional scan zoom of 2.52× to achieve maximum magnification (0.11 µm per voxel).

### Experimental design and statistical analysis

For patch-clamp electrophysiology, *n* = 38 neurons from *n* = 24 mice (12 females/12 males, P59–78) from *n* = 13 different litters were used. Only one recorded neuron per brain section was used, to prevent ambiguity in morphological renderings. Analysis was performed in Clampfit 10. Input resistance was calculated from the linear slope of the voltage responses to the first 3–4 hyperpolarizing current steps. Time constant was calculated from an exponential curve to a 10 pA hyperpolarizing current. Sag ratio was defined as the ratio of the steady-state voltage to the peak negative voltage reached during a hyperpolarization step. Action potential threshold, latency, peak, amplitude, spike duration, upslope, and downslope were analyzed from the first elicited action potential (at the rheobase current). Threshold was measured as the first point with dV/dt >20 mV/ms. Latency was defined as the time between the beginning of the current step and threshold. Amplitude was calculated as the difference between peak voltage and threshold. Spike duration was measured as the width of the spike halfway between threshold and peak. Upslope/downslope ratio was defined as the ratio between the maximum upslope and downslope, which were calculated as the largest and smallest values (respectively) of dV/dt during the action potential. Burst index was measured using the voltage response to a depolarizing current 50 pA greater than rheobase for each neuron. It was calculated as 1 − *F*0 / *F*1, where *F*0 is the peak–peak frequency of the first two spikes of the spike train and *F*1 is the peak–peak frequency of the last two spikes.

**Figure 1.**
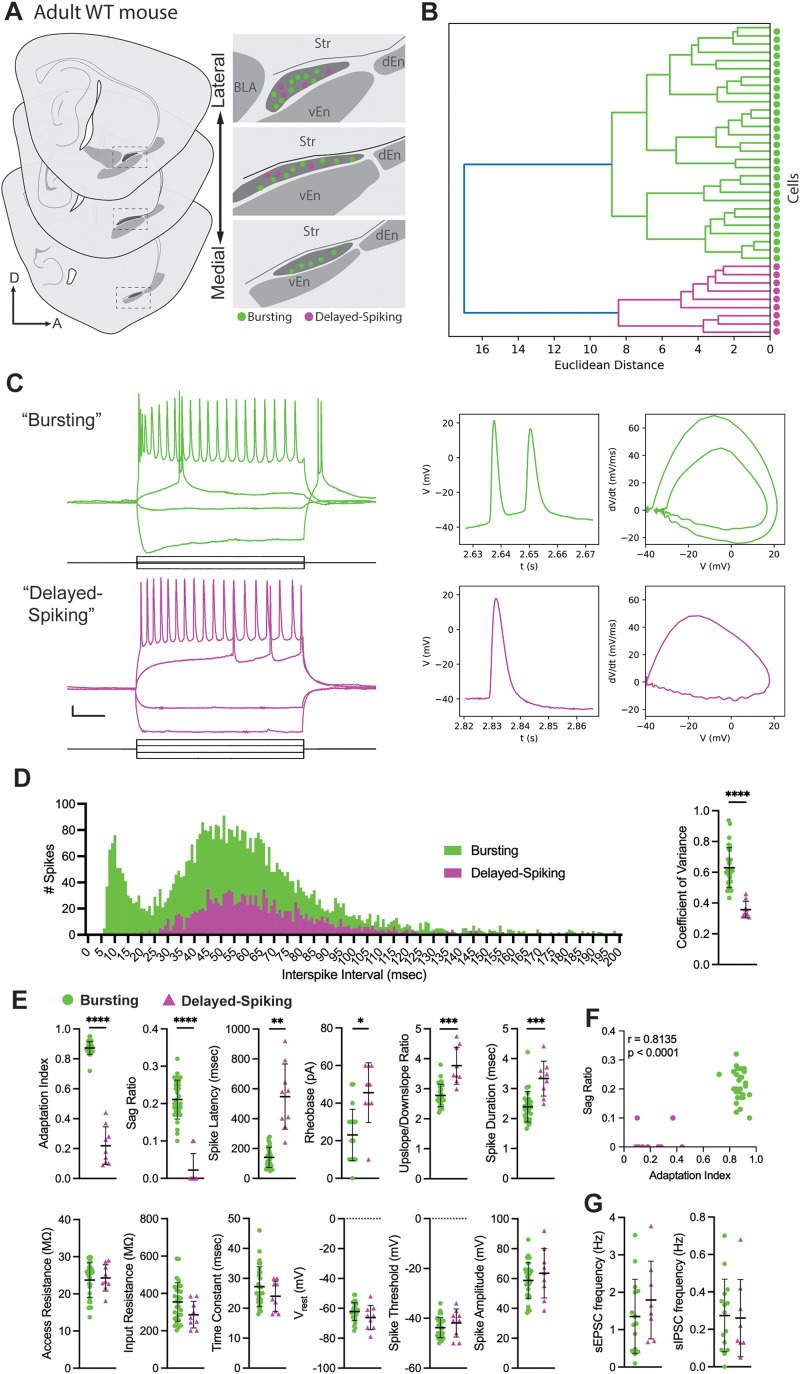
Electrophysiological subtypes in the adult mouse paralaminar amygdala. ***A***, Patch-clamp electrophysiology was performed in sagittal sections from adult (P59–78) WT mice targeting neurons across the PL. Right, Mediolateral map of PL, with approximate locations of recorded neurons. ***B***, An agglomerative hierarchical clustering dendrogram depicts two clusters of cells (green and magenta) separated by a Euclidean distance of 17. ***C***, Left, Neurons within each cluster exhibited stereotyped action potential patterns in response to 1 s current injections. Right, Representative rheobase action potentials and phase–plane plots for each cluster. ***D***, Left, Frequency histogram of interspike intervals, summed for all neurons within each cluster. Right, Coefficient of variance of interspike interval histograms. ***E***, Comparisons between subtypes of each individual electrical parameter used for clustering. ***F***, Correlation plot of adaptation index and sag ratio for all recorded neurons. ***G***, Frequencies of spontaneous excitatory (sEPSC) and inhibitory (sIPSC) postsynaptic currents. Extended Data Figure 1-1 shows analysis of maturation stage and sex differences in adult PL neuron electrophysiological properties. Calibrations in ***C***: 10 mV (vertical) and 200 ms (horizontal). All data are mean ± SD. *p < 0.05, **p < 0.01, ***p < 0.001, ****p < 0.0001. Abbreviations: BLA, basolateral complex; dEn, dorsal endopiriform; vEn, ventral endopiriform; Str, striatum.

Following analysis, agglomerative hierarchical clustering (with Ward linkage and Euclidean distance) was performed using the matplotlib package (Python; Fig. 1). The following input variables were used for clustering: resting membrane potential, input resistance, time constant, and sag ratio, as well as action potential latency, rheobase, threshold, amplitude, duration, burst index, and upslope/downslope ratio. Capacitance, action potential peak voltage, upslope, and downslope were excluded from clustering because of their redundancy to other input variables (time constant, capacitance, amplitude, and upslope/downslope ratio). Clustering results were used to compare each individual physiological and morphological parameter between subtypes. For these comparisons (Fig. 1*D*,*E*), multiple Welch *t* tests were employed, with the Holm–Sidak post hoc multiple-comparisons correction. To analyze burst properties across electrophysiological subtypes (Fig. 1*D*), a frequency histogram was generated using the interspike intervals for all action potentials across current injections in all cells. Next, using the frequency distribution for each cell, a coefficient of variance was calculated and compared across clusters. Action potential phase–plane plots (Fig. 1*C*) were generated using the matplotlib package (Python), using code available on the spikesandbursts online blog (Cabrera-Garcia, 2022).

10.1523/ENEURO.0119-24.2024.f1-1 Figure 1-1 **Maturational stages and sex differences in morpho-electric properties of adult (P59-78) mouse PL neurons.** (A) T-tests comparing electrophysiological properties of adult PL neurons to the juvenile PL neurons in our previously published dataset (Alderman et al. 2024). (B) Threshold and spike duration plotted against input resistance. Dotted lines represent the cutoffs defining mature neurons in the juvenile PL from Alderman et al. (2024). (C) Input resistance, spike duration, and threshold plotted against animal age. (D) Volcano plot of q values (FDR-corrected p values, see methods) comparing sex differences in morphological and electrical properties of adult PL neurons. Horizontal dotted line represents an alpha cutoff of 0.05. Download Figure 1-1, TIF file.

To compare electrophysiological properties between the adult and juvenile PL (Extended Data Fig. 1-1), the adult dataset collected in this study was compared with a previously published juvenile PL dataset (Alderman et al., 2024), which was collected via the same equipment, protocol, and investigator. Multiple Welch *t* tests were used with Holm–Sidak post hoc multiple-comparisons correction.

**Figure 2.**
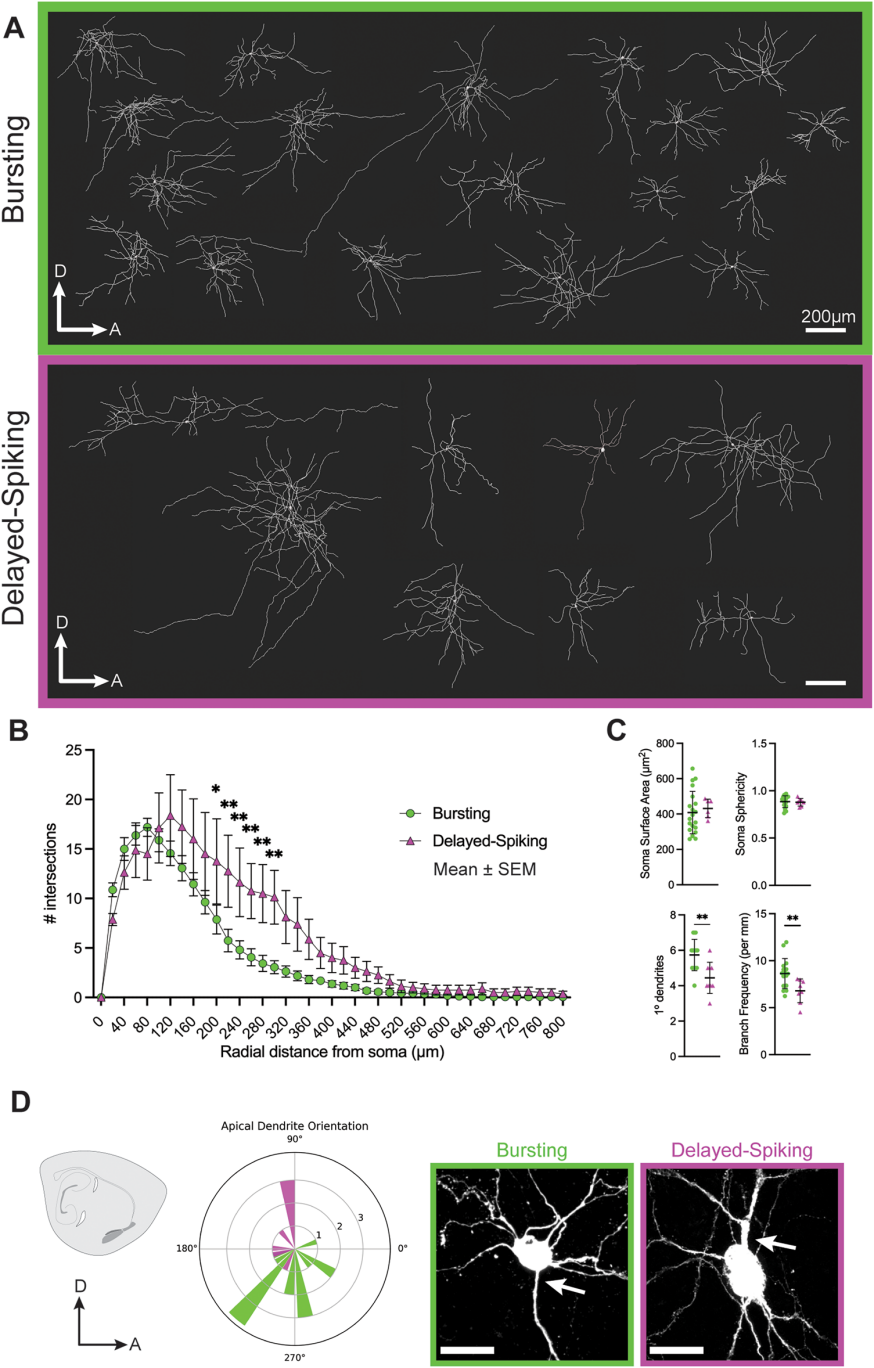
Bursting and delayed-spiking PL neurons possess distinct morphologies. Following patch-clamp recordings, post hoc biocytin staining and morphological reconstructions (***A***) were performed on bursting (green) and delayed-spiking (magenta) neurons. ***B***, Number of Sholl intersections in each subtype at 40 µm radial intervals. ***C***, Comparisons between subtypes of soma surface area and sphericity, number of primary dendrites (dendrites directly emanating from the soma), and branch frequency (number of branches per filament distance). Data are mean ± SD. ***D***, Polar histogram of the orientation angle of apical dendrites (left), and examples of apical dendrites in each subtype (right, arrows). Scale bars: ***A***, 200 µm; ***D***, 20 µm. *p < 0.05, **p < 0.01.

Morphological analysis (Fig. 2) was performed in Imaris 10. Each neuron was reconstructed using the “Filaments” tool in the 3D view, and the soma was rendered using the “Surfaces” tool. Sholl analysis was performed using concentric radii of 20 µm each. Branch frequency was calculated by dividing the number of branch points by the total filament length for each neuron. For apical dendrite orientation analysis, apical dendrites were identified by their large diameter and length and opposing orientation relative to other dendrites. For each apical dendrite, the “segment orientation angle” measurement was recorded from the Imaris “Filaments” tool. A polarity histogram was generated using the matplotlib package (python).

Evaluation of sex differences in morpho-electric criteria (Extended Data Fig. 1-1) was performed using *z*-scores of each electrophysiological and morphological variable. *z*-scores were obtained using the fit_transform() function in the matplotlib package (Python). This transformed data was analyzed using multiple Welch *t* tests with the Holm–Sidak post hoc multiple-comparisons correction.

**Figure 3.**
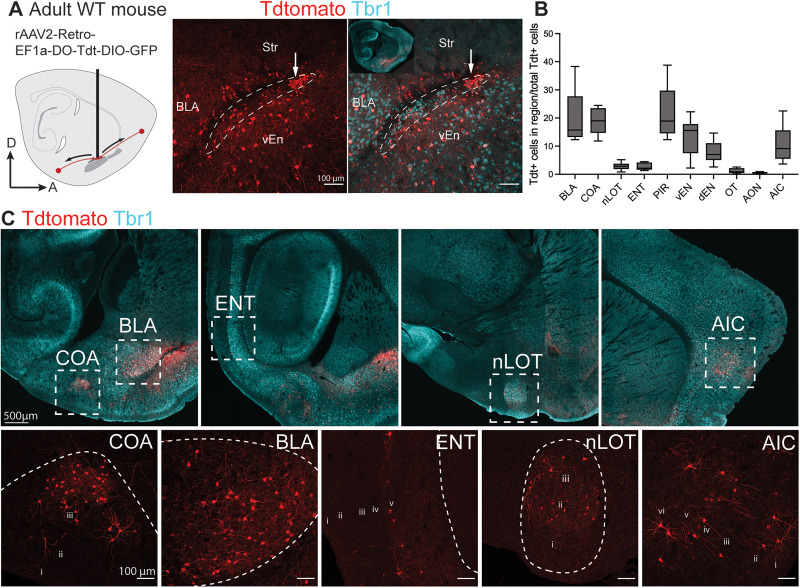
Retrograde viral labeling reveals putative regions of input to the PL. ***A***, rAAV2-Retro-EF1a-DO-Tdt-DIO-GFP was injected to the PL of P50–64 WT mice. White arrow indicates injection site. ***B***, Quantification of tdTomato+ labeled neurons within each brain region as a percentage of total labeled neurons for each mouse. ***C***, Input neurons located within the cortical and basolateral amygdala, entorhinal cortex, nucleus of the lateral olfactory tract, and agranular insular cortex. See Extended Data Figure 3-1 for qualitative assessment of injection targeting. Abbreviations: BLA, basolateral complex; dEn, dorsal endopiriform; vEn, ventral endopiriform; COA, cortical amygdala; ENT, entorhinal cortex; nLOT, nucleus of the lateral olfactory tract; AIC, agranular insular cortex.

**Figure 4.**
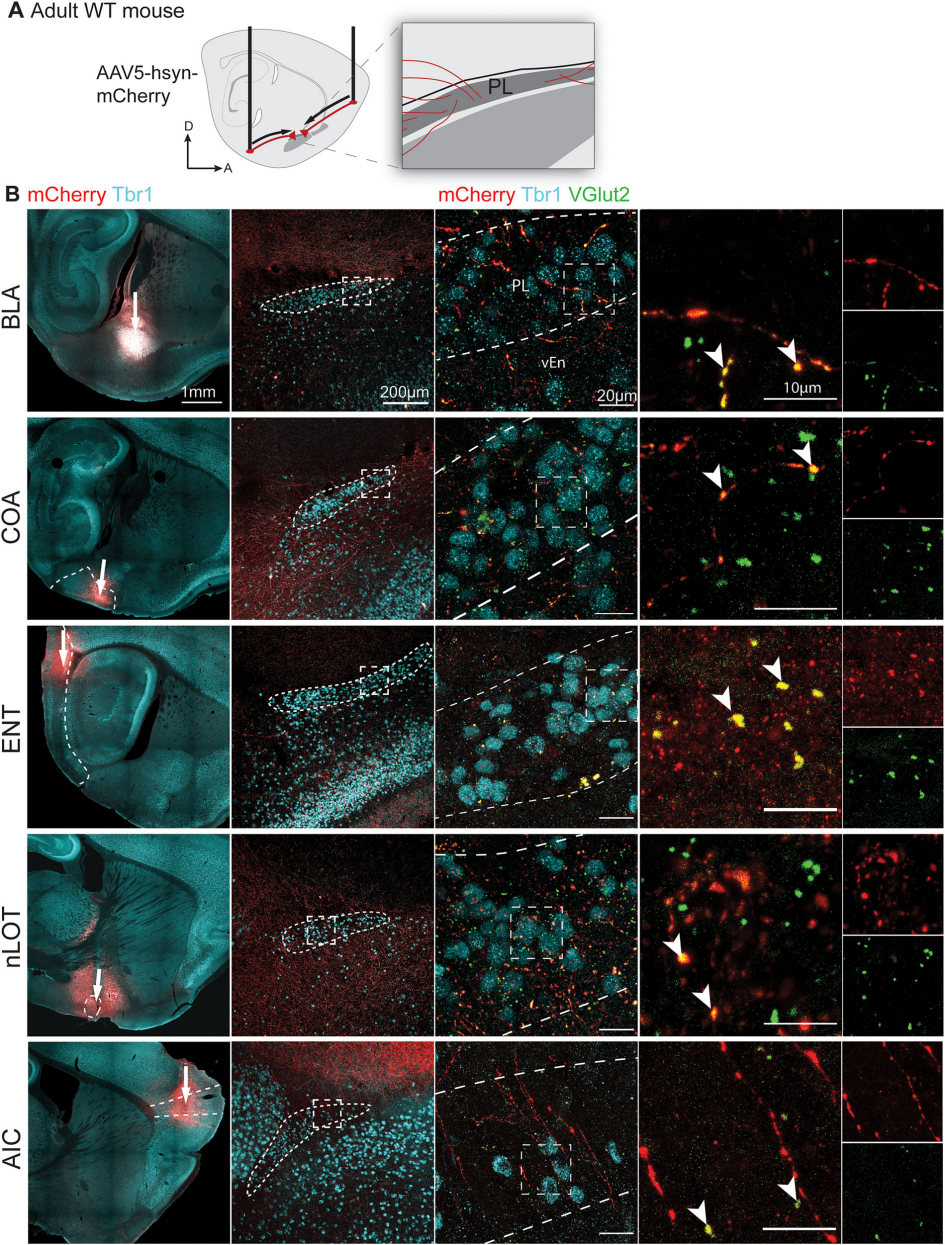
Anterograde viral labeling of inputs to the PL. ***A***, Major regions of input uncovered in Figure 3 were infected with AAV5-hsyn-mCherry in P54–68 WT mice. ***B***, Injections to either the BLA, COA, ENT, nLOT, or AIC (left, white arrows indicate injection sites) and labeled fibers (mCherry) within the PL following each injection (center). Labeled fibers colocalized with VGlut2+ putative excitatory presynaptic terminals (far right). Extended Data Figure 4-1 shows qualitative injection targeting scores.

**Figure 5.**
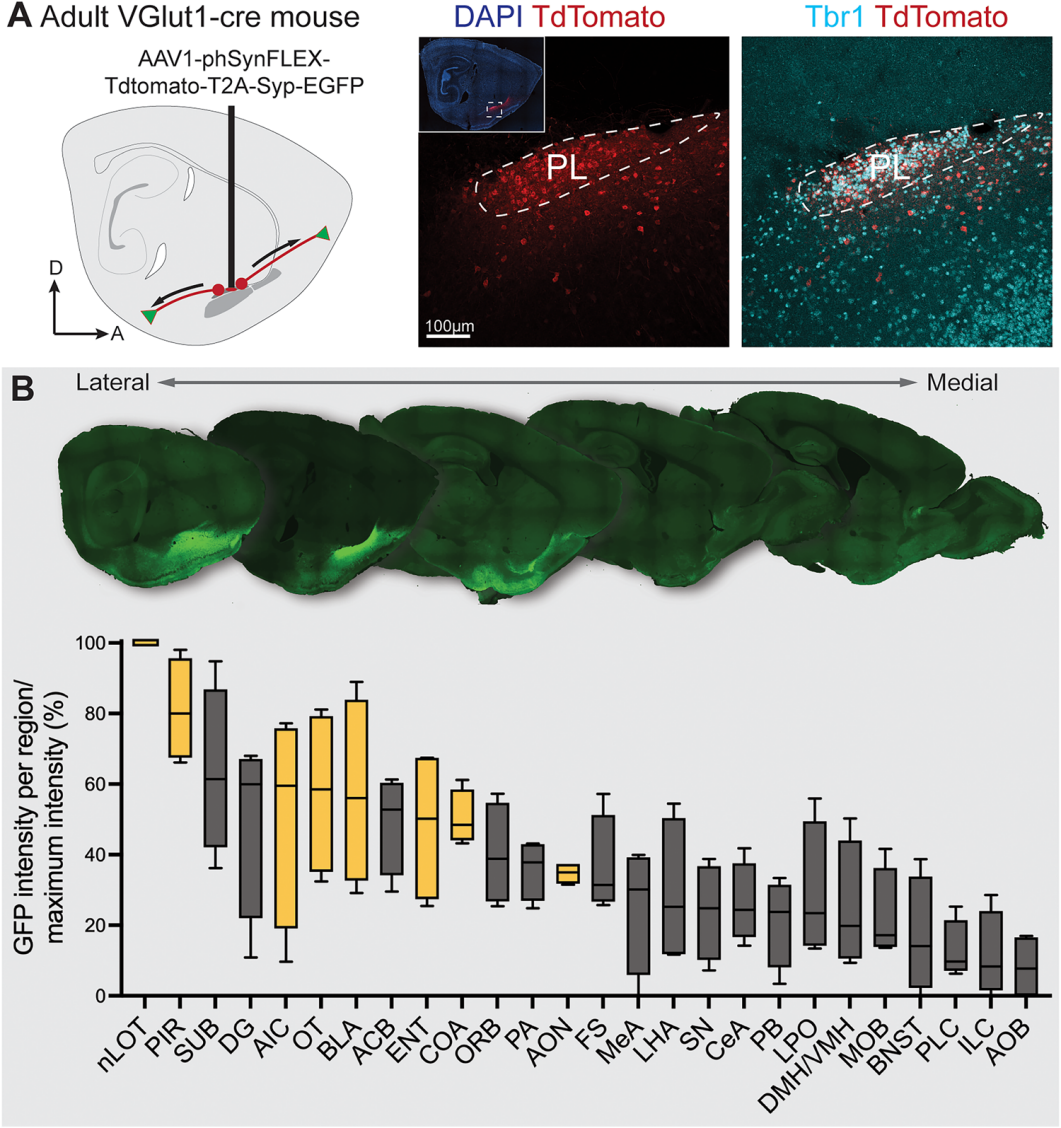
Anterograde viral tracing revealing the output projections from the adult mouse PL. ***A***, AAV1-phSyn1(S)-FLEX-tdtTomato-T2A-Syp-EGFP-WPRE was injected to the PL of P54–112 *VGlut1-cre* mice. ***B***, Example sagittal sections (at 600 µm intervals) and quantified mean intensity of Syp-EGFP labeling within brain regions. EGFP intensity was normalized in each mouse, with 100 and 0% corresponding to the highest and lowest observed intensities respectively. Yellow boxes indicate regions with reciprocal input projections to the PL. Abbreviations: See Table 1. Extended Data Figure 5-1 shows qualitative injection targeting scores.

**Figure 6.**
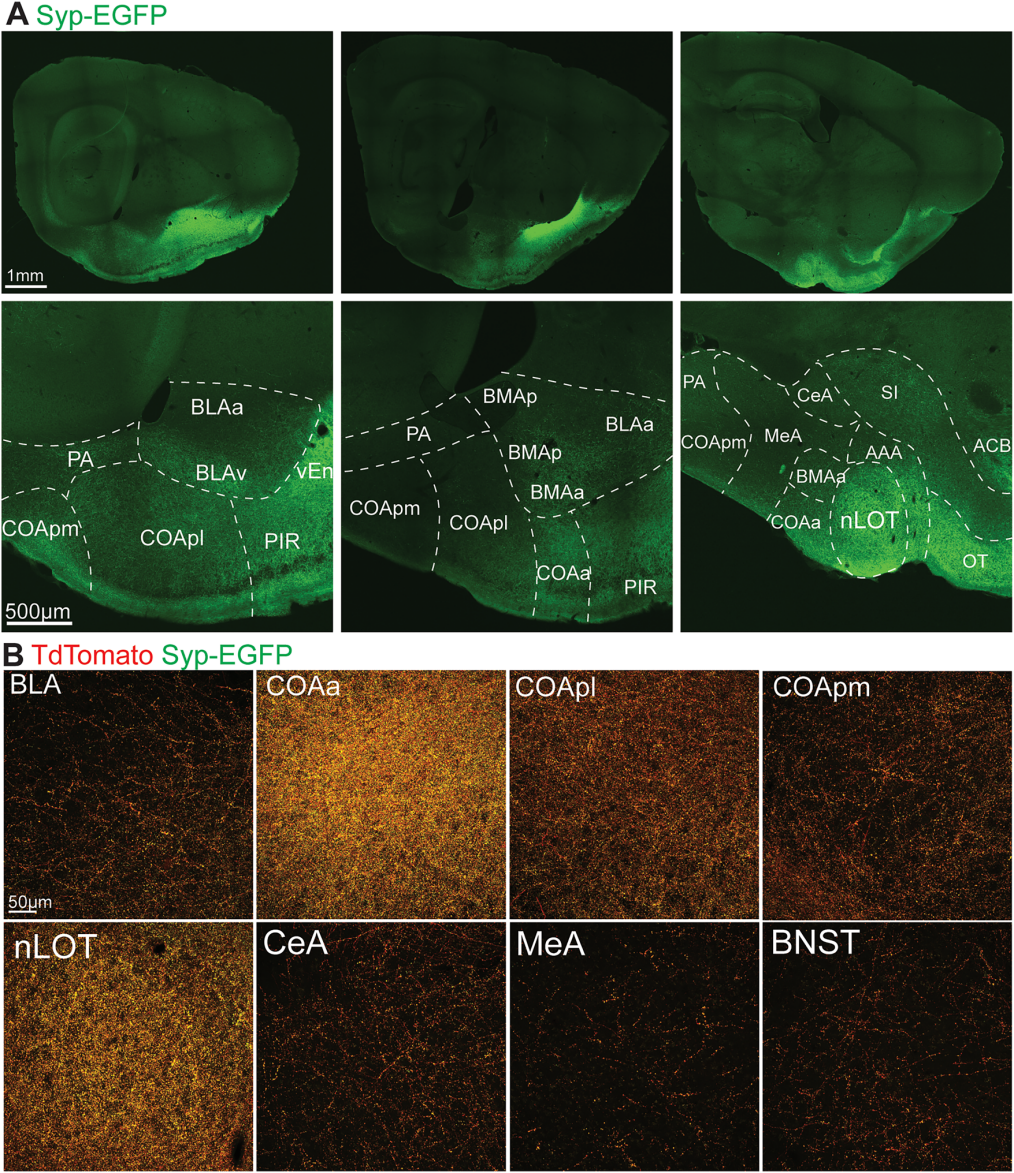
Anterograde viral labeling of PL outputs in the amygdala. ***A***, Example Syp-EGFP labeling across the amygdala. ***B***, High magnification images of PL output fibers (tdTomato) and synaptic terminals (EGFP) within amygdala subnuclei. Abbreviations: See Table 1.

**Figure 7.**
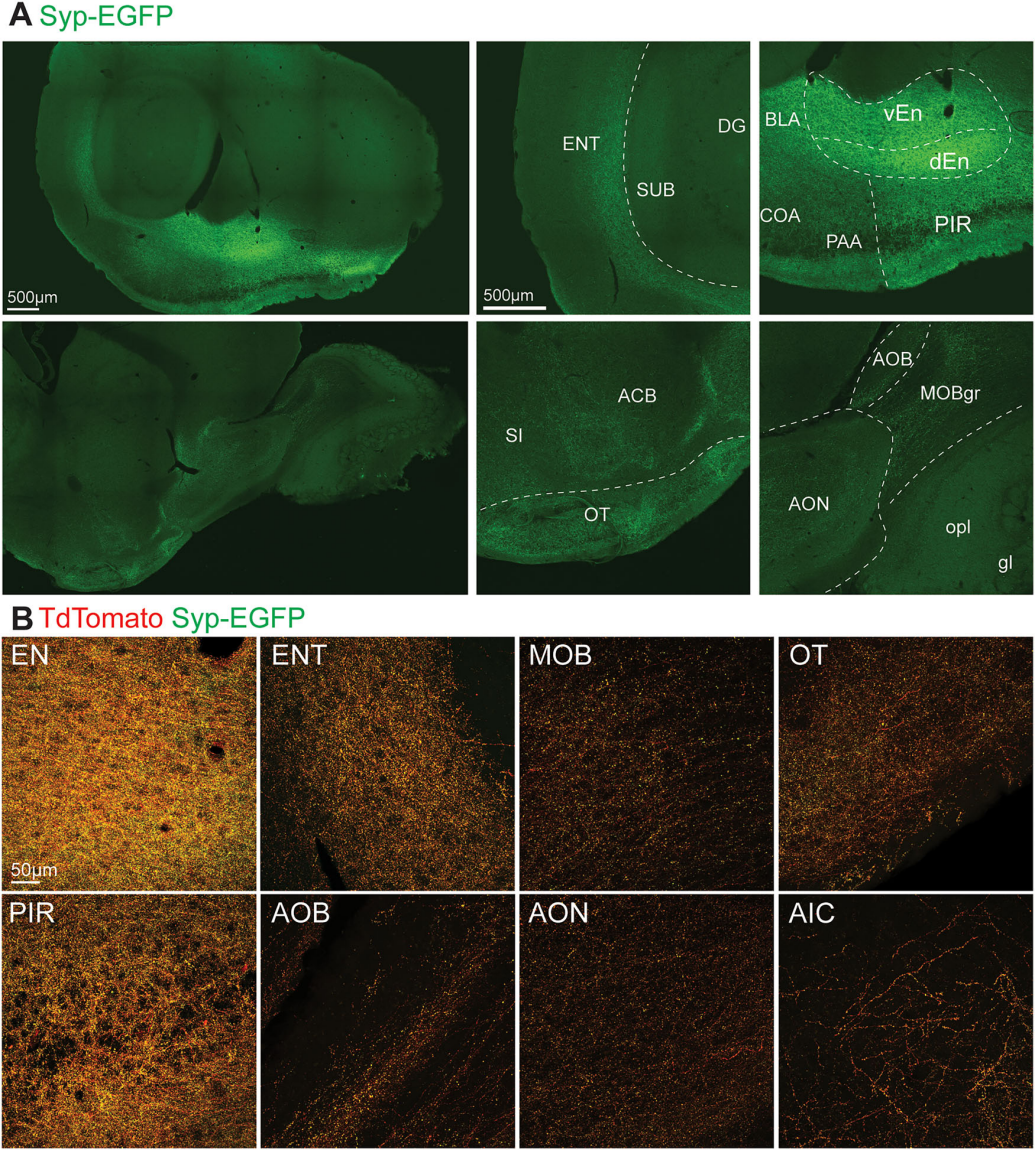
Anterograde viral labeling of PL outputs in the olfactory network. ***A***, Example Syp-EGFP labeling across olfactory network regions. ***B***, High magnification images of PL output fibers (tdTomato) and synaptic terminals (EGFP) within olfactory network regions. Abbreviations: See Table 1.

**Figure 8.**
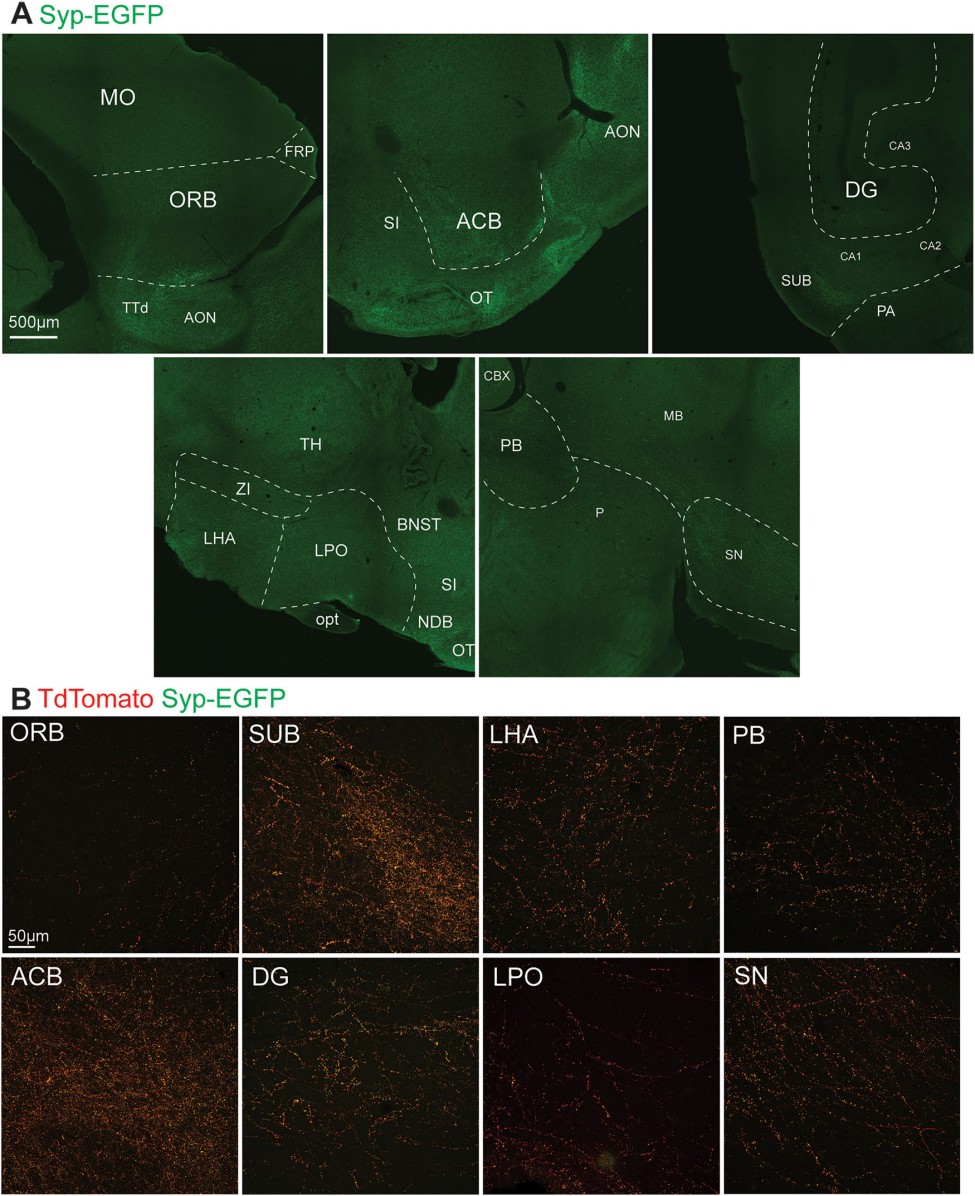
Anterograde viral labeling of PL outputs in frontal, striatal, hippocampal, hypothalamic, and brainstem regions. ***A***, Example Syp-EGFP labeling. ***B***, High magnification images of PL output fibers (tdTomato) and synaptic terminals (EGFP) within frontal, striatal, hippocampal, hypothalamic, and brainstem regions. Abbreviations: See Table 1.

For retrograde tracing of PL inputs (Fig. 3), *n* = 6 mice (2 males, 4 females) with successful PL targeting were analyzed. To control for variable transduction across mice, labeled neurons in each input region were not directly compared between mice, but instead compared as a percentage of total input neurons within each mouse. For anterograde tracing of input sources projecting to the PL (Fig. 4), *n* = 3 mice per input source were analyzed [2 females, 1 male for the cortical amygdala (COA), nucleus of the lateral olfactory tract (nLOT), entorhinal cortex (ENT), agranular insular cortex (AIC) and 2 males, 1 female for the basolateral complex (BLA)]. Analysis was done by confocal visualization of labeled fibers within the PL colocalized with VGlut2 expression. For anterograde tracing of output targets emanating from the PL (Figs. 5–8), *n* = 4 mice (3 males, 1 female) with successful targeting were analyzed. Analysis was done using Nikon NIS-Elements software using a 10× large image stitch of an entire slide. For each output target region, multiple ROIs were drawn across sections, and the average intensity of the FITC channel (Syp-EGFP) was measured. The ventral (vEN) and dorsal (dEN) endopiriform regions were excluded from analysis due to viral leakage from the adjacent PL. Intensity values within each mouse were normalized to percentages, using 0% as the lowest observed intensity and 100% as the highest observed intensity (GraphPad Prism 10).

10.1523/ENEURO.0119-24.2024.f3-1 Figure 3-1 **Injection targeting of retrograde virus to the adult mouse PL** * Scoring criteria: •• 50% of PL targeted, ••• 75% of PL targeted, •••• > 90% of PL targeted. - no infected neurons, + 1-10 infected neurons, ++ 10-20 infected neurons (per 50  µm section) Download Figure 3-1, TIF file.

10.1523/ENEURO.0119-24.2024.f4-1 Figure 4-1 **Injection targeting of anterograde virus to adult mouse PL input sources** * Scoring criteria: •• 50% of region targeted, ••• 75% of region targeted, •••• > 90% of region targeted. - no infected neurons, + 1-10 infected neurons, ++ 10-20 infected neurons (per 50  µm section). Download Figure 4-1, TIF file.

10.1523/ENEURO.0119-24.2024.f5-1 Figure 5-1 **Injection targeting of anterograde virus to the adult mouse PL** * Scoring criteria: • 25% of PL targeted, •• 50% of PL targeted, ••• 75% of PL targeted, •••• > 90% of PL targeted .- no infected neurons, + 1-10 infected neurons, ++ 10-20 infected neurons (per 50  µm section). Download Figure 5-1, TIF file.

**Table 1.** List of abbreviations

AAA	Anterior amygdala area	LPO	Lateral preoptic area
ACB	Nucleus accumbens	MB	Midbrain
AIC	Agranular insular cortex	MeA	Medial amygdala
AOB	Accessory olfactory bulb	MO	Somatomotor cortex
AON	Anterior olfactory nucleus	MOB	Main olfactory bulb
BLA	Basolateral complex of the amygdala	gr	granule layer
BNST	Bed nucleus of the stria terminalis	opl	outer plexiform layer
CBX	Cerebellar cortex	gl	glomerular layer
CeA	Central amygdala	nLOT	Nucleus of the lateral olfactory tract
COA	Cortical amygdala	opt	Optic tract
COAa	Anterior cortical amygdala	ORB	Orbitofrontal cortex
COApl	Posterolateral cortical amygdala	OT	Olfactory tubercle
COApm	Posteromedial cortical amygdala	P	Pons
DG	Dentate gyrus of the hippocampus	PA	Posterior amygdala
DMH	Dorsomedial hypothalamus	PB	Parabrachial nucleus of the pons
EN	Endopiriform cortex/nucleus	PIR	Piriform cortex
vEN	Ventral endopiriform	PL	Paralaminar nucleus of the amygdala
dEN	Dorsal endopiriform	PLC	Prelimbic cortex
ENT	Entorhinal cortex	SI	Substantia innominata
FRP	Frontal pole	SN	Substantia nigra
FS	Fundus of striatum	Str	Striatum
ILC	Infralimbic cortex	SUB	Subiculum
ITC	Intercalated cells of the amygdala	TT	Tenia tecta
LHA	Lateral hypothalamic area	VMH	Ventromedial hypothalamus

## Results

### Electrophysiological subtypes of adult PL neurons

The adult mouse PL contains excitatory neurons, but their diversity is unstudied. To investigate the electrical properties of these cells, we performed patch-clamp electrophysiological recordings from individual PL neurons in adult (P59–78) C57/Bl6 mice (Fig. 1). We measured voltage responses to 1 s hyperpolarizing and depolarizing current steps of increasing magnitude in *n* = 38 neurons (from *n* = 24 mice) sampled from multiple levels across the PL (Fig. 1*A*). To generate an unbiased representation of physiological diversity in adult PL neurons, we performed unsupervised agglomerative hierarchical clustering of recorded cells, using 11 intrinsic electrical parameters (see Materials and Methods). The resulting dendrogram segregated cells into two main clusters (Fig. 1*B*), which corresponded to different action potential firing patterns (Fig. 1*C*). All neurons within the larger cluster (*n* = 29 cells) exhibited initial bursts of 2–3 action potentials during depolarization and anomalous rectification during hyperpolarization. In the smaller cluster, all neurons (*n* = 9) exhibited extended spike latencies (between 241 and 791 ms) during depolarization, and no rectification during hyperpolarization. To further investigate these dichotomous spike patterns, we plotted a frequency histogram of the interspike intervals for all recorded cells (Fig. 1*D*). One cluster exhibited a bimodal distribution resembling an intrinsically bursting neuron (Nowak et al., 2003; Latuske et al., 2015), with the first mode at 10 ms corresponding to the initial bursting depolarizations and the second at 51 ms corresponding to tonic activity later in the spike trains. The other cluster displayed a unimodal distribution at 45 ms, resembling tonic (nonbursting) activity. The coefficient of variance was significantly greater in the cluster with burst activity (*t*_(32.73)_ = 9.039; *p* < 0.0001; Welch's *t* test with Holm–Sidak correction). Based on these stereotyped patterns, we labeled these neurons “bursting” and “delayed-spiking” neurons.

To better understand the differences between bursting and delayed-spiking PL neurons, we performed unpaired *t* tests of each physiological parameter used for hierarchical clustering individually (Fig. 1*E*). There were no significant differences in access resistance (*t*_(17.49)_ = 0.395; *p* = 0.746), passive membrane properties (RMP *t*_(10.89)_ = 1.36, *p* = 0.5308; input resistance *t*_(19.50)_ = 2.243, *p* = 0.2008; time constant *t*_(17.44)_ = 1.55, *p* = 0.5296; multiple Welch *t* tests with Holm–Sidak correction), action potential threshold (*t*_(10.71)_ = 0.95; *p* = 0.5988), or amplitude (*t*_(10.68)_ = 0.81; *p* = 0.5988). There were statistically significant differences in adaptation index (*t*_(8.593)_ = 15.26; *p* < 0.0001), spike latency (*t*_(8.49)_ = 5.487; *p* = 0.0047), and sag ratio (*t*_(15.66)_ = 10.70; *p* < 0.0001; multiple Welch *t* tests with Holm–Sidak correction) between subtypes, which reflected the distinct properties we observed in action potential patterns (Fig. 1*C*). Further analysis revealed that adaptation index and sag ratio were strongly positively correlated (*r* = 0.8135; *p* < 0.0001; Pearson's *r*) with complete divergence in these properties across subtypes (Fig. 1*F*). In addition to these differences, cluster analysis revealed that bursting neurons exhibit significantly lower rheobase (*t*_(11.9)_ = 3.82; *p* = 0.0171), spike duration (*t*_(12.03)_ = 4.36; *p* = 0.0083), and upslope/downslope ratio (*t*_(9.897)_ = 4.54; *p* = 0.0087; multiple Welch *t* tests with Holm–Sidak correction). Together, these observations define two distinct electrophysiological subtypes of neurons in the adult mouse PL.

In a subset of neurons, we measured spontaneous synaptic input activity (*n* = 23; Fig. 1*G*). There was no significant difference in the frequency of excitatory (sEPSC) and inhibitory (sIPSC) postsynaptic currents between bursting and delayed-spiking neurons (sEPSC *t*_(21)_ = 0.994, *p* = 0.8314; sIPSC *t*_(21)_ = 0.156, *p* = 0.7946; unpaired *t* tests). These observations suggest that there are no differences in the frequency of synaptic input between bursting and delayed-spiking PL subtypes.

Based on our prior work showing that PL neurons develop along a delayed trajectory, we next evaluated whether the electrophysiological subtypes we observed were due to different stages of maturation of PL neurons. Our previous studies indicated that by molecular criteria, ∼90% of neurons in the adult (P60) mouse PL are mature (Dcx−, NeuN+; Alderman et al., 2024). Further, in our previous recordings of juvenile ages (P21–28), we observed that electrical maturation of PL neurons is defined by a decrease in input resistance, hyperpolarization of threshold, and reduction in spike duration (Alderman et al., 2024). Compared with this juvenile dataset, adult PL neurons exhibit significantly decreased input resistance (*t*_(26.14)_ = 6.031; *p* < 0.0001), threshold (*t*_(41.44)_ = 2.406; *p* = 0.0370), and spike duration (*t*_(27.43)_ = 2.500; *p* = 0.0370; unpaired Welch *t* tests with Holm–Sidak correction), indicating that PL neurons continue to undergo electrophysiological maturation between juvenile and adult ages (Extended Data Fig. 1-1*A*). To investigate possible differences in maturation stage between subtypes, we plotted this combination of features in adult PL neurons (Extended Data Fig. 1-1*B*). This revealed that, in contrast to the juvenile PL neurons assessed in our prior studies, adult PL neuron physiological properties do not fall on a maturational continuum. Further, across the adult age range we recorded (P59–78), none of the criteria that define juvenile PL neuron maturational stage (input resistance, threshold, spike duration) correlated with animal age (input resistance *r* = −0.2; threshold *r* = −0.02; spike duration *r* = −0.07; Pearson's *r*; Extended Data Fig. 1-1*C*). This data indicates that electrophysiological differences in adult PL subtypes are not based on different maturational stages but instead represent two distinct subtypes.

### Adult PL neuron electrophysiological subtypes have distinct morphologies

We next examined whether there were morphological differences between bursting and delayed-spiking PL neurons (Fig. 2). During patch-clamp recordings, *n* = 18/29 bursting neurons and *n* = 8/9 delayed-spiking neurons were filled with biocytin, and we post hoc reconstructed their morphologies in 275-µm-thick sections. The anatomical location and orientation of each reconstructed cell was also determined. The delayed-spiking neurons we recorded were primarily located in lateral PL sections with none observed in the medial PL, whereas the bursting neurons we recorded were distributed throughout the PL (Fig. 1*A*). In each subtype, the reconstructed neurons exhibited substantial variety in morphology (Fig. 2*A*), similar to our previous findings in the juvenile PL where morphological complexity varied independently of physiological maturity (Alderman et al., 2024).

To determine whether bursting and delayed-spiking neurons exhibit differences in soma size, shape, or dendritic properties, we analyzed these properties using Imaris imaging software. Sholl analysis revealed that delayed-spiking neurons exhibited a greater number of Sholl intersections from 200 to 300 µm radii (200 µm *p* = 0.0413; 220 µm *p* = 0.0038; 240 µm *p* = 0.0059; 260 µm *p* = 0.0077; 280 µm *p* = 0.0033; 300 µm *p* = 0.0029; mixed-effects ANOVA with Sidak correction; Fig. 2*B*). The somas of bursting and delayed-spiking neurons were of similar size (*t*_(24)_ = 0.459; *p* = 0.6504) and sphericity (*t*_(24)_ = 0.245; *p* = 0.8082), but bursting neurons had a greater number of primary dendrites (*t*_(24)_ = 3.46; *p* = 0.0022) as well as a higher branch frequency (*t*_(24)_ = 2.83; *p* = 0.0098; unpaired *t* tests; Fig. 2*C*). Finally, we examined the proximal angles of the apical dendrites in each reconstructed neuron and found that apical dendrites were preferentially oriented toward the pial surface in bursting neurons and away from the pial surface in delayed-spiking neurons (Fig. 2*D*). Thus, adult PL neurons display diverse morphologies with bursting and delayed-spiking subtypes exhibiting significant differences in dendritic architecture.

The amygdala is a sexually dimorphic structure. To investigate whether neurons in the adult PL exhibited sex differences, we compared the physiological and morphological features collected from male (*n* = 12) and female (*n* = 12) mice. Across all features, we observed no significant differences between sexes (multiple Welch *t* tests with Holm–Sidak correction; Extended Data Fig. 1-1*D*).

From these combined observations, we conclude that the excitatory neurons of the adult mouse PL comprise two subtypes defined by distinct action potential firing patterns and morphologies. In both subtypes, we recorded a similar frequency of spontaneous synaptic input. We next investigated the sources of this input to the PL.

### The mouse PL receives major input from the main olfactory network and amygdala basolateral complex

To uncover the sources of neuronal input to the mouse PL, we performed unilateral PL injections in adult WT mice (P50–64, *n* = 6) with the retrograde virus *rAAV2-retro-EF1a-DO-TdTomato-DIO-EGFP* (Addgene #37120; Fig. 3*A*). Following 2–3 weeks of incubation, we observed tdTomato+ neurons in multiple regions within and outside the amygdala (Fig. 3*B*,*C*). We quantified the relative locations of these cells within each injected brain by normalizing the number of tdTomato+ cells in each region to the total number infected per mouse (Fig. 3*B*). Across all experiments, labeled neurons were observed in multiple nuclei of the main olfactory network: anterior olfactory nucleus (AON), piriform cortex (PIR), olfactory tubercle (OT), cortical amygdala (COA), nucleus of the lateral olfactory tract (nLOT), entorhinal cortex (ENT), and agranular insular cortex (AIC), as well as in the amygdala basolateral complex (BLA; Fig. 3*B*).

The relatively small size of the PL makes it challenging to specifically target without viral spread to the nearby intercalated cell clusters (ITCs), external capsule, and ventral endopiriform (vEN). We qualitatively assessed the extent of this spread in our injections and did not detect differences in traced input regions based on viral spread (Extended Data Fig. 3-1).

Although these observations were consistent across injections, many of the regions we observed also project to the nearby piriform and endopiriform cortex (Neville and Haberly, 2004; Majak and Moryś, 2007). Therefore, we next investigated whether these putative input sources send projections specifically into the PL. To accomplish this, we selected a subset of regions that contained a substantial proportion of putative input neurons (>1% of total tdT+ cells). In these putative major input regions (BLA, COA, nLOT, ENT, and AIC), we performed unilateral injections in adult WT mice (P57–67) in separate experiments with the anterograde virus *rAAV5-hsyn-mCherry-WPRE* ((Addgene #114472; Extended Data Fig. 4-1). Following 3 weeks of incubation, brains were sectioned at 50 µm and stained with Tbr1 to label the PL, mCherry to reveal infected cells and projections, and VGlut2 to label excitatory synaptic terminals (Fig. 4). We observed mCherry+ fibers emanating from each injected region to the PL. In all experiments, these fibers contained puncta that colocalized with the glutamatergic presynaptic marker VGlut2. Together, these findings indicate that the PL receives excitatory neuronal input from the basolateral complex, cortical amygdala, nucleus of the lateral olfactory tract, entorhinal cortex, and agranular insular cortex.

### The adult mouse PL projects reciprocally to its input sources and to the hippocampus, striatum, hypothalamus, and brainstem

We next investigated which brain regions are targeted by PL output projections. The mouse PL expresses the glutamatergic marker Slc17a7 (VGlut1), distinguishing it from the neighboring inhibitory ITCs and ventral striatum (Alderman et al., 2024). We performed injections in the PL of P54–112 *VGlut1-ires-cre* transgenic mice (Jax #037512, *n* = 4) with the anterograde virus *AAV1-phSyn-FLEX-Tdtomato-T2A-Syp-EGFP* (Addgene #51509), which, in the presence of cre recombinase, labels infected neurons with tdTomato and synaptic terminals with EGFP (Fig. 5*A*; Oh et al., 2014). As the nearby basolateral complex and piriform/endopiriform cortex also contain VGlut1-expressing cells, we qualitatively scored their degree of infection (Extended Data Fig. 5-1).

Following 3 weeks of incubation, we performed sagittal sectioning of whole brains and conducted a brain-wide survey of GFP labeling (putative synaptic terminals), quantifying it in each labeled brain region (Fig. 5*B*). To evaluate whether the GFP signal intensity observed corresponded to synaptic terminals labeled by viral infection, we verified the colocalization of GFP with tdTomato+ labeled fibers in every observed target region (Figs. 6–8). Across all regions, synaptic terminals (GFP) colocalized with fibers (tdTomato).

Strong GFP signal was observed in all regions revealed as inputs in our retrograde tracing experiments (Fig. 4). The strongest signal across mice was in the nLOT and dense terminals were also observed in the basolateral (BLA) and cortical (COA) nuclei of the amygdala (Fig. 6). In the COA, terminals were densest in the anterior subregion. Investigation of the olfactory network confirmed wide targeting of all structures by labeled terminals, including input sources (ENT, EN, PIR, OT, AON, AIC), as well as the main and accessory olfactory bulbs (MOB, AOB; Fig. 7). In addition to the amygdala and olfactory network, the hippocampal subiculum (SUB) and dentate gyrus (DG) exhibited labeling, as well as the nucleus accumbens (ACB; Figs 5*B*, 8). Finally, labeled fibers traveled to the hypothalamus and into the brainstem (Fig. 8), with putative synaptic terminals present in the lateral and medial hypothalamus, substantia nigra, and parabrachial nucleus (Fig. 5*B*). Notably we did not observe labeling in other brain regions (e.g., nongustatory isocortex, thalamus, caudate and putamen, medulla).

Together, these results indicate that the outputs of the PL emanate primarily to olfactory, amygdala, hippocampal, and striatal structures, with additional projections to hypothalamus and brainstem. This suggests that the PL may influence olfactory, affective, and contextual learning, as well as autonomic and visceral states.

## Discussion

Using a combination of electrophysiological, morphological reconstruction, and viral based circuit mapping, we characterized the intrinsic properties of mature PL neurons and mapped their input/output connectivity. From these studies, we identified bursting and delayed-spiking neuronal subtypes in the adult mouse PL which show differences in firing properties and dendritic arborization. We found that the PL is reciprocally connected to olfactory regions, with additional outputs primarily to valence processing and memory structures. Our findings provide critical novel understanding of this understudied population and are essential to elucidate the role of this region in complex brain and behavior changes that unfold during the transition from adolescence to adulthood and in the adult brain.

Action potential patterns are a common distinguishing feature of neuronal subtypes. PL subtypes are differentiated by the presence or absence of bursting, a property which frequently defines divergent circuit functions in both vertebrates and invertebrates (Lisman, 1997; Krahe and Gabbiani, 2004; Zeldenrust et al., 2018). One mechanism for bursting is the backpropagation of an initial spike into the proximal dendrites, activating dendritic conductances that cause an afterdepolarization (ADP); in the presence of a low-threshold spike, this ADP crosses threshold and results in a burst (Häusser et al., 2000; Izhikevich, 2007). The greater dendritic complexity in the bursting subgroup is consistent with this putative mechanism. Further, in physiological conditions, bursting may reflect the spatial summation of multiple inputs to different dendrites (Poirazi and Mel, 2001; Zeldenrust et al., 2018). One consequence of a burst is synaptic release potentiation at axon terminals, thereby increasing signaling to postsynaptic targets (Swadlow and Gusev, 2001). In the PL, therefore, the bursting subtype may exhibit stronger transmission to downstream neurons than the delayed spiking subtype. Meanwhile, delayed-spiking neurons require larger and longer current injections to reach threshold, indicating that this subtype may be less excitable in vivo. Together, this suggests that PL neuronal subtypes may constitute distinct coding elements as a direct consequence of their intrinsic electrophysiological differences.

Despite the key dendritic differences between bursting and delayed-spiking neurons, both subtypes exhibit within-group variability. In prior work in the juvenile PL, we observed that some neurons with immature physiological and molecular features possess surprisingly complex morphologies. Thus, dendritic complexity appears only partly influenced by physiological subtype and cell maturity. However, two potential technical sources of the morphological variance we observe are the possible uneven biocytin filling across patched neurons and differences in transection of neurites during slice preparation. Both reduce the recoverable fraction of the total dendritic tree. Thus, we focused on metrics which are robust to these confounds. Soma size and shape and primary dendrite number do not depend on distal neurites which may be lost. Similarly, branch frequency (branches per mm), which displayed relatively low variability in our dataset, is robust despite uneven filling because both branch number and total dendritic length are affected. Uncovering the factors that might influence PL neuron morphology, such as different input and output connectivity or hormonal influences, and how they correlate with subtypes, are important future directions.

Interestingly, the finding of two excitatory neuron subtypes within the adult PL is similar to the heterogeneity of neuronal types found in other late-maturing regions. In the late-maturing piriform cortex, two types of principal neurons exist, defined by the presence or absence of bursting as well as morphological correlates (Suzuki and Bekkers, 2006). Further, these subtypes are differentially connected to olfactory and cortical afferents (Suzuki and Bekkers, 2011). However, specifically whether late-maturing piriform neurons comprise both subtypes is unknown. In the neurogenic dentate gyrus of the rodent, newly born neurons mature into granule cells (GCs) with greater morphological complexity than typically developed GCs (Cole et al., 2020). A lesser studied dentate population are semilunar GCs, which exhibit earlier birth and different morpho-electric properties than GCs (Save et al., 2019; Gupta et al., 2020). In addition, although they are not late-maturing, the excitatory principal neurons in the basolateral complex have also been divided into two groups, which exhibit bursting and delayed-spiking features similar to the PL (Washburn and Moises, 1992; Rainnie et al., 1993; Paré et al., 1995). Further, these subgroups are differentially active in vivo (Paré and Gaudreau, 1996). This indicates that the subtypes found in the adult mouse PL recapitulate patterns of heterogeneity present in other brain regions, and that bursting and delayed-spiking PL neurons likely exhibit differences in input/output circuitry and/or functional roles.

Input and output tracing results reveal that the adult mouse PL is reciprocally connected to the main olfactory network and basolateral complex. The main olfactory network is defined by the receipt of direct input from the olfactory bulb, and all regions were revealed as both PL input sources and output projections. The olfactory bulb itself was not a source of direct input to the PL but it was a target of PL output projections, which were localized to the granule layer. In contrast, the accessory olfactory network (AOB, MeA, BNST) was not a source of PL input, receiving comparatively sparse PL output projections, suggesting that the PL is not involved in innate behavioral responses to pheromones and odorants. Finally, bidirectional input–output connectivity to the basolateral complex, which encodes innate and learned valence of polysensory stimuli, may confer the attractiveness or repulsiveness of olfactory cues. In previous studies, we observed c-*fos* responses in PL neurons following exposure to both appetitive and aversive odors (Alderman et al., 2024); this activity may be conferred by reciprocal BLA connectivity. Thus, the PL receives second-order sensory information from olfactory network regions, likely integrating this with valence processing (via BLA connections) and feeds this information back to the olfactory network. Importantly, the inputs we observed may not be exhaustive, as some studies indicate that retro-AAV tracing shows evidence of tropism for cortical excitatory neurons (Chatterjee et al., 2018; Zhu et al., 2019). While we observed comparatively low levels of inhibitory synaptic activity in recorded neurons (Fig. 1*G*), it remains possible that nuclei populated by inhibitory output neurons (e.g., striatum) may send projections to the PL not detected in our experiments.

Beyond its reciprocal connectivity to olfactory regions, output projections from the PL further highlight its putative processing of valence (via basolateral amygdala outputs), a possible role in memory (via hippocampal outputs), and influence over visceral and autonomic states (via pontine and hypothalamic outputs). A key question is whether these connections are overlapping or exclusive to PL subpopulations. Many PL outputs are similar to those of the piriform cortex, which projects to the olfactory and prefrontal cortex, cortical amygdala, and entorhinal cortex, as well as the striatum and hypothalamus (Price et al., 1991; Johnson et al., 2000; Neville and Haberly, 2004; Bekkers and Suzuki, 2013). These output connections appear spatially and functionally organized (Y. Chen et al., 2022), contrasting the long-held belief that they are stochastic (Miyamichi et al., 2011; Schaffer et al., 2018). One possibility is that the PL is similarly organized into distinct circuit motifs, which further extend to its additional outputs. This would place the PL as a hub which associates specific olfactory cues with valence information, possibly supporting specific behavioral responses, such as approach/avoidance, autonomic arousal, and memory encoding. Because these interpretations are based on morphological observations of anatomically traced input and outputs, further functional dissection of PL connectivity will be highly informative.

In primates, tracing studies indicate input fibers to the PL from the ventral hippocampus and lateral amygdala (Pitkänen and Amaral, 1998; Fudge et al., 2012), and output projections from the PL targeting the basal and central amygdala as well as the ventral striatum (Fudge et al., 2002; Fudge and Tucker, 2009). Our connectivity findings in the mouse are broadly concordant with these findings, except for the ventral hippocampus which did not have detected inputs to the PL in mouse, and the dense connectivity of the olfactory cortex to the mouse PL. The latter may reflect the greater reliance of olfaction in rodents compared with primates.

In humans, alterations in the number or molecular signatures of PL neurons are seen in multiple neuropsychiatric disorders. In autism spectrum disorder, PL neurons exhibit alterations in transcripts relevant to maturation, and dysfunctional PL development correlates with a lack of amygdala growth into adulthood (Avino et al., 2018, Sorrells et al., 2019). The adult PL is smaller in volume in military veterans with posttraumatic stress disorder as well as adults with temporal lobe epilepsy, compared with healthy controls (Morey et al., 2020; Ballerini et al., 2022). Therefore, the PL neuron subtypes and circuitry may be sensitive to genetic and environmental insults and may confer key circuitry affected by neurological and psychiatric disorders. Further studies in humans, and relevant animal models of these and other disorders, will be critical to understand the relevance of the PL in various neuropsychiatric conditions.

In summary, we reveal that the adult mouse PL contains two neuronal subtypes, is reciprocally connected to the olfactory network, and projects widely to striatal, hippocampal, hypothalamic, and brainstem targets. These findings highlight the significance of the PL across species as a late-maturing amygdala subregion that may serve as a key nexus supporting the behavioral transition to adulthood. Given that synaptic input, mature intrinsic identities, and behavioral responsivity of the PL emerge between juvenile and adult ages, an important next step is to track these changes during adolescence, as well as determine the interrelationships between the development of inputs, intrinsic properties, and learning.

## Synthesis

Reviewing Editor: Julie Bakker, University of Liege Decisions are customarily a result of the Reviewing Editor and the peer reviewers coming together and discussing their recommendations until a consensus is reached. When revisions are invited, a fact-based synthesis statement explaining their decision and outlining what is needed to prepare a revision will be listed below. The following reviewer(s) agreed to reveal their identity: NONE. Note: If this manuscript was transferred from JNeurosci and a decision was made to accept the manuscript without peer review, a brief statement to this effect will instead be what is listed below.

synthesis: 2 reviewers have evaluated the ms and both find the study interesting and important. However, they ask for some nuancing in the interpretation of the results since there are some limitations in the methodology used (e.g. the viral approach used for retrograde tracing).

Reviewer 1:

In this study, Saxon and colleagues use a combination of ex vivo electrophysiology, neuronal morphology analysis and viral anatomical tracing to further characterize the recently discovered paralaminar nucleus of the amygdala (PL) in the adult mouse. The authors use unsupervised hierarchical clustering to identify two separate classes of neurons, a bursting- and a delayed spiking type, which have a partially segregated spatial distribution within the nucleus and different dendritic complexities. Finally, the authors use retrograde and anterograde viral tools to characterize the inputs and outputs of PL neurons with particular emphasis on their communication with the olfactory network.

This research is timely and interesting, providing valuable information on a mostly unexplored nucleus of the amygdala of the mouse. Here are some comments that will hopefully help to improve the rigor of the study and the interpretability of the results.

Main points:

1. My main concern is regarding the viral approach used for retrograde tracing. While retroAAVs are an excellent tool for the retrograde tracing of cortical structures, they tend to display very low efficiency on multiple subcortical structures, particularly, but not exclusively, those rich in GABAergic projection neurons. In our hands, retroAAVs failed to trace projections from structures such as the MeApd, VMH and BNST (while these projections can be traced anterogradely from the other end or retrogradely with other viral tools). Similar results have been already reported by other labs (for instance, see Zhu Xutao et al., 2020 Neuroscience Bulletin, Figure 3). Given this caveat, the interpretation of the anatomical results on the areas that are reciprocally connected versus the areas that are not should be carefully reconsidered.

Ideally, the retroAAV results should be contrasted with another tracing method, either classic tracer (Fluorogold, CTB) or viral (herpes simplex virus, see Lo et al., 2019 PNAS). Alternatively, this limitation in the tracing approach should be clearly acknowledged in the discussion and the interpretation of these results toned down.

2. Regarding the implementation of the hierarchical clustering analysis: The following input variables were used for clustering: resting membrane potential, input resistance, time constant, and sag ratio, as well as action potential latency, rheobase, threshold, amplitude, duration, burst index, and upslope/downslope ratio. Capacitance, action potential peak voltage, upslope, and downslope were excluded from clustering.

What is the rationale in this choice? How robust is the presence of these clusters to the inclusion of these excluded parameters?

3. Was access resistance measured and compared across the clustered groups? This could have an impact in parameters such as action potential peak, and possibly, in the clustering analysis.

On a similar note, was there any bridge balance compensation applied? These experimental details should be reported in the methods section of the manuscript.

4. Is there any medio-lateral distribution of the inputs reported in Figure 4? Given that the identified clusters of PL neurons are partially segregated throughout the mediolateral axis, it would be interesting to understand whether the different anatomically defined inputs show any spatial segregation. This point was specifically motivated because the example levels of the PL displayed in the second column do not seem to be at a fixed mediolateral level.

Minor points:

5. In the introduction, is stated that approx. 80% of PL neurons express the marker Tbr1. Interestingly, the clustering analysis shows that approx. 75% of neurons belong to one of the clusters. Did the authors attempt to match cluster identity to the expression of this marker given the resemblance in proportion?

6. sIPSCs are recorded at -40mV, which according to figure suppl. 1 should be beyond the threshold to fire for several of these neurons. Does this result in escaped spikes in these voltage clamp recordings? How was this dealt with for analysis?

7. Phase plots of action potentials would likely be illustrative of several of the changes reported in figure 1.

8. There is a typo in line 279. mV should be MΩ.

Reviewer 2:

This descriptive study into the cellular subtypes and connectivity of the paralaminar nucleus of the amygdala represents an important step towards understanding how this important brain region may function within neural circuits, and will be of interest to the field. In general, the work has been carefully and painstakingly carried out, with convincing results. I have a few comments.

A key finding is the discovery of two subtypes of excitatory neurons in the PL. This is based on a number of electrophysiological parameters, but one of the most important drivers (indeed giving the manuscript names for each group) is the bursting firing behaviour present in one group. Given this, it would be good to: 1) quantify this directly - in fact, in the methods it states that a bursting index is calculated, but in Figure 1 and the main text it is referred to as an adaptation index. Indeed, the way it has been calculated is more appropriate for an adaptation index. There are ways to more directly quantify bursting, and it would be great to show this; 2) show the bursting firing more convincingly in the figure, for example using traces taken when injecting only just above suprathreshold current.

I was going to suggest plotting some of the principal components, which I think can help to illustrate the point better than just the dendrogram, but in fact I then found something similar in the figures at the end of the text! Unfortunately, the embedded figures have not been updated, and nor have some of the figure legends (for Fig1) or the main text (Figs1/2) - this needs amending.

In the main text (lines 341-2) it states 'labeled neurons were observed in multiple nuclei of the main olfactory network (AON...)' but the graph (3B) suggests almost none in the AON. It would be interesting to see quantification in other non-olfactory areas for comparison. Similarly, for the output mapping, are there areas that show no signal?

To be clear, while of course there are endless ways these findings could be enhanced by additional experiments (e.g. functional confirmation of connectivity, relating connectivity to subtypes, postsynaptic labelling to more convincingly show synaptic connections, etc.) I do not believe these are necessary or called for at this stage, nor would be likely to change the message of the paper. It might be worth nuancing the conclusions a bit to make it clear that 'connectivity' is based only on limited morphological investigation?

Minor points:

There are quite a few places where abbreviations appear without being spelt out. While there is a table, I think in some of these cases it would be helpful for readers to still write them out first:

Methods, lines 226, 231

Figure 1 - define abbreviations from 1A in legend

Figure 3 - ditto.

Line 356 - define ITCs and vEN.

Figure 5 - too many here to define in legend, but could refer to Table 1 for abbreviations.

Line 69 - I realise that 'circuital' is a word, but not sure it is normally used in this way!

## Author Response

Dear eNeuro Editorial Staff, We are pleased that our manuscript, "Neuronal subtypes and connectivity of the adult mouse paralaminar amygdala," has been judged potentially suitable for publication in eNeuro. We also appreciate the constructive comments from the editor and reviewers. We have made a number of changes based on the helpful input from reviewers.

Reviewer 1:

Critique 1. Concerns with the retroAAV displaying very low efficiency on multiple subcortical structures, particularly, but not exclusively, those rich in GABAergic projection neurons. Given this caveat, the interpretation of the anatomical results on the areas that are reciprocally connected versus the areas that are not should be carefully reconsidered. Ideally, the retroAAV results should be contrasted with another tracing method, either classic tracer (Fluorogold, CTB) or viral (herpes simplex virus, see Lo et al., 2019 PNAS). Alternatively, this limitation in the tracing approach should be clearly acknowledged in the discussion and the interpretation of these results toned down.

Response: Though not reported in the initial characterization of retroAAV (Tervo et al., Neuron, 2016), we recognize that labeling using retroAAV may be cell type and region-specific dependent. However, in a recent study from our group (Prakash et al., J. Comp Neurol., 2024), we successfully employed retroAAV to study long range input into the amygdala, indicating that this tool is useful for amygdala labeling. Nevertheless, given the potential caveats, we have updated the discussion to acknowledge the tropism observed with retroAAV tracing leaving open the possibility of additional connections from inhibitory regions.

Critique 2. Regarding the implementation of the hierarchical clustering analysis: The following input variables were used for clustering: resting membrane potential, input resistance, time constant, and sag ratio, as well as action potential latency, rheobase, threshold, amplitude, duration, burst index, and upslope/downslope ratio. Capacitance, action potential peak voltage, upslope, and downslope were excluded from clustering.

What is the rationale in this choice? How robust is the presence of these clusters to the inclusion of these excluded parameters? Response: In the clustering analysis, each input variable was weighted equally, and we focused on both passive and active electrical properties. The rationale for exclusion of some variables was due to their redundancy with other variables. For example, action potential peak voltage is the sum of the threshold and amplitude, which are both included as input variables. Similarly, capacitance was excluded due to redundancy with time constant and input resistance. Upslope and downslope were excluded due to redundancy with the upslope/downslope ratio. We have updated the text of the methods to better describe this rationale. Finally, we performed hierarchical clustering with all variables included, and found no difference in the presence of these two clusters, highlighting the robustness of our observations.

Critique 3. Was access resistance measured and compared across the clustered groups? This could have an impact in parameters such as action potential peak, and possibly, in the clustering analysis.

On a similar note, was there any bridge balance compensation applied? These experimental details should be reported in the methods section of the manuscript.

Response: We thank the reviewer for this important insight. We measured access resistance for all cells and excluded cells with access resistance >30MΩ. No bridge balance compensation was applied. We have updated the methods to describe these details. We have also added a new comparison of access resistance across the clustered groups, which was nonsignificant (p=0.745, see new Figure 1E).

Critique 4. Is there any medio-lateral distribution of the inputs reported in Figure 4? Given that the identified clusters of PL neurons are partially segregated throughout the mediolateral axis, it would be interesting to understand whether the different anatomically defined inputs show any spatial segregation. This point was specifically motivated because the example levels of the PL displayed in the second column do not seem to be at a fixed mediolateral level.

Response: We did not observe medio-lateral distribution of input fibers within the PL. While we did observe that fibers from the BLA and AIC appeared to reach the PL via the striatum, and other sources (COA, ENT, nLOT) traveled via ventral pallial structures (displayed in Figure 4B), once fibers reached the PL they appeared distributed throughout the nucleus. Deeper dissection of major inputs using more sophisticated viral and optogenetic tools and their role in driving circuit function and behavior is a major focus of our future work.

Minor points:

Critique 5. In the introduction, is stated that approx. 80% of PL neurons express the marker Tbr1. Interestingly, the clustering analysis shows that approx. 75% of neurons belong to one of the clusters. Did the authors attempt to match cluster identity to the expression of this marker given the resemblance in proportion? Response: Our attempt to match cluster identity to Tbr1 expression was limited by variable antibody signal in biocytin post hoc filled cells as compared to immune-staining on tissue. However, a subset of recorded neurons was successfully co-labeled with Tbr1, including neurons within each cluster. Further, all recorded neurons displayed tonic or intrinsically-bursting spike patterns, spiny dendritic trees, and distally projecting axons, resembling cortical excitatory neurons.

Critique 6. sIPSCs are recorded at -40mV, which according to figure suppl. 1 should be beyond the threshold to fire for several of these neurons. Does this result in escaped spikes in these voltage clamp recordings? How was this dealt with for analysis? Response: While we observed spikes during the beginning of these voltage clamp experiments, the sIPSC recording period was performed during time periods with no escaped spikes due to voltage gated Na channel inactivation.

Critique 7. Phase plots of action potentials would likely be illustrative of several of the changes reported in figure 1.

Critique 8. There is a typo in line 279. mV should be MΩ.

Response: This is corrected.

Reviewer 2:

Critique 1. A key finding is the discovery of two subtypes of excitatory neurons in the PL. This is based on a number of electrophysiological parameters, but one of the most important drivers (indeed giving the manuscript names for each group) is the bursting firing behaviour present in one group. Given this, it would be good to: 1) quantify this directly - in fact, in the methods it states that a bursting index is calculated, but in Figure 1 and the main text it is referred to as an adaptation index. Indeed, the way it has been calculated is more appropriate for an adaptation index. There are ways to more directly quantify bursting, and it would be great to show this; 2) show the bursting firing more convincingly in the figure, for example using traces taken when injecting only just above suprathreshold current.

Response: We appreciate this important feedback. We have updated figure 1 with further quantification to more clearly demonstrate the presence of bursting which drives subtype differences. While many bursting quantification strategies exist, many apply to the detection of in vivo stochastic bursting, rather than current clamp experiments. In addition to the adaptation index calculation already in the manuscript, we have added a new histogram of inter-spike interval data for each cluster which clearly shows a bimodal distribution (as well as significantly greater coefficient of variance) in the bursting subtype (new Figure 1D). Finally, we show a single suprathreshold voltage trace to clearly show the bursting pattern in one subtype (new Figure 1C).

Critique 2. I was going to suggest plotting some of the principal components, which I think can help to illustrate the point better than just the dendrogram, but in fact I then found something similar in the figures at the end of the text! Unfortunately, the embedded figures have not been updated, and nor have some of the figure legends (for Fig1) or the main text (Figs1/2) - this needs amending.

Response: We have now corrected this.

Critique 3. In the main text (lines 341-2) it states 'labeled neurons were observed in multiple nuclei of the main olfactory network (AON...)' but the graph (3B) suggests almost none in the AON. It would be interesting to see quantification in other non-olfactory areas for comparison. Similarly, for the output mapping, are there areas that show no signal? Response: In our quantification of retrograde-traced input neurons (Figure 3), we counted every labeled input neuron observed across the brain and noted their locations. This included a few neurons per animal within the AON. While these were sparse, we still included them as part of our total. There were numerous brain regions with no input neurons, and they were not included in the graph despite being surveyed. The approach was similar for output mapping - many brain regions showed no signal, and thus were not included in the graph.

Critique 4. It might be worth nuancing the conclusions a bit to make it clear that 'connectivity' is based only on limited morphological investigation? Response: We have updated the discussion to clarify that our connectivity interpretations are based on anatomical and morphological observations.

Minor points:

There are quite a few places where abbreviations appear without being spelt out. While there is a table, I think in some of these cases it would be helpful for readers to still write them out first:

Methods, lines 226, 231 Figure 1 - define abbreviations from 1A in legend Figure 3 - ditto.

Line 356 - define ITCs and vEN.

Figure 5 - too many here to define in legend, but could refer to Table 1 for abbreviations.

Line 69 - I realise that 'circuital' is a word, but not sure it is normally used in this way! Response: Thank you for these helpful suggestions, we have updated the text to incorporate these changes.

## References

[B1] Ai J-Q, Luo R, Tu T, Yang C, Jiang J, Zhang B, Bi R, Tu E, Yao Y-G, Yan X-X (2021) Doublecortin-expressing neurons in Chinese tree shrew forebrain exhibit mixed rodent and primate-like topographic characteristics. Front Neuroanat 15:727883. 10.3389/fnana.2021.727883 34602987 PMC8481370

[B2] Alderman PJ, et al. (2024) Delayed maturation and migration of excitatory neurons in the juvenile mouse paralaminar amygdala. Neuron 112:574–592. 10.1016/j.neuron.2023.11.010 38086370 PMC10922384

[B3] Ascoli GA, et al. (2008) Petilla terminology: nomenclature of features of GABAergic interneurons of the cerebral cortex. Nat Rev Neurosci 9:557–568. 10.1038/nrn2402 18568015 PMC2868386

[B4] Avino TA, Barger N, Vargas MV, Carlson EL, Amaral DG, Bauman MD, Schumann CM (2018) Neuron numbers increase in the human amygdala from birth to adulthood, but not in autism. Proc Natl Acad Sci U S A 115:3710–3715. 10.1073/pnas.1801912115 29559529 PMC5889677

[B5] Ballerini A, et al. (2022). Amygdala subnuclear volumes in temporal lobe epilepsy with hippocampal sclerosis and in non-lesional patients. Brain Commun 4:fcac225. 10.1093/braincomms/fcac225 36213310 PMC9536297

[B6] Bekkers JM, Suzuki N (2013) Neurons and circuits for odor processing in the piriform cortex. Trends Neurosci 36:429–438. 10.1016/j.tins.2013.04.005 23648377

[B7] Benedetti B, et al. (2020) Functional integration of neuronal precursors in the adult murine piriform cortex. Cereb Cortex 30:1499–1515. 10.1093/cercor/bhz181 31647533 PMC7132906

[B8] Benedetti B, Couillard-Despres S (2022) Why would the brain need dormant neuronal precursors? Front Neurosci 16:877167. 10.3389/fnins.2022.877167 35464307 PMC9026174

[B9] Benedetti B, Reisinger M, Hochwartner M, Gabriele G, Jakubecova D, Benedetti A, Bonfanti L, Couillard-Despres S (2023) The awakening of dormant neuronal precursors in the adult and aged brain. Aging Cell e13974. 10.1111/acel.13974 37649323 PMC10726842

[B10] Borzello M, Ramirez S, Treves A, Lee I, Scharfman H, Stark C, Knierim JJ, Rangel LM (2023) Assessments of dentate gyrus function: discoveries and debates. Nat Rev Neurosci 24:502–517. 10.1038/s41583-023-00710-z 37316588 PMC10529488

[B11] Cabrera-Garcia D (2022) Patch-clamp data analysis in python: action potentials | spikes and bursts. Spikes and Bursts. Available at: https://spikesandbursts.wordpress.com/2022/05/03/patch-clamp-analysis-python-action-potentials/#phase-plane-plot

[B12] Chaker Z, Segalada C, Kretz JA, Acar IE, Delgado AC, Crotet V, Moor AE, Doetsch F (2023) Pregnancy-responsive pools of adult neural stem cells for transient neurogenesis in mothers. Science 382:958–963. 10.1126/science.abo5199 37995223

[B13] Chareyron LJ, Lavenex PB, Amaral DG, Lavenex P (2012) Postnatal development of the amygdala: a stereological study in macaque monkeys. J Comp Neurol 520:1965–1984. 10.1002/cne.23023 22173686 PMC4043192

[B14] Chatterjee S, et al. (2018) Nontoxic, double-deletion-mutant rabies viral vectors for retrograde targeting of projection neurons. Nat Neurosci 21:638–646. 10.1038/s41593-018-0091-7 29507411 PMC6503322

[B15] Chen Y, Chen X, Baserdem B, Zhan H, Li Y, Davis MB, Kebschull JM, Zador AM, Koulakov AA, Albeanu DF (2022) High-throughput sequencing of single neuron projections reveals spatial organization in the olfactory cortex. Cell 185:4117–4134.e28. 10.1016/j.cell.2022.09.038 36306734 PMC9681627

[B16] Chen S, Xu H, Dong S, Xiao L (2022) Morpho-electric properties and diversity of oxytocin neurons in paraventricular nucleus of hypothalamus in female and male mice. J Neurosci 42:2885–2904. 10.1523/JNEUROSCI.2494-21.2022 35197315 PMC8985873

[B17] Cole JD, Espinueva DF, Seib DR, Ash AM, Cooke MB, Cahill SP, O’Leary TP, Kwan SS, Snyder JS (2020) Adult-born hippocampal neurons undergo extended development and are morphologically distinct from neonatally-born neurons. J Neurosci 40:5740–5756. 10.1523/JNEUROSCI.1665-19.2020 32571837 PMC7380968

[B18] Crosby EC, Humphrey T (1941) Studies of the vertebrate telencephalon II. The nuclear pattern of the anterior olfactory nucleus, tuberculum olfactorium and the amygdaloid complex in adult man. J Comp Neurol 74:309–352. 10.1002/cne.900740209

[B19] deCampo DM, Fudge JL (2012) Where and what is the paralaminar nucleus? A review on a unique and frequently overlooked area of the primate amygdala. Neurosci Biobehav Rev 36:520–535. 10.1016/j.neubiorev.2011.08.007 21906624 PMC3221880

[B20] Denoth-Lippuner A, Jessberger S (2021) Formation and integration of new neurons in the adult hippocampus. Nat Rev Neurosci 22:223–236. 10.1038/s41583-021-00433-z 33633402

[B21] Dieni CV, Gonzalez JC, Overstreet-Wadiche L (2019) Multifaceted circuit functions of adult-born neurons. F1000Res 8:1998. 10.12688/f1000research.20642.1 PMC6880266 31824650

[B22] Fudge JL (2004) Bcl-2 immunoreactive neurons are differentially distributed in subregions of the amygdala and hippocampus of the adult macaque. Neuroscience 127:539–556. 10.1016/j.neuroscience.2004.05.019 15262342 PMC2435199

[B23] Fudge JL, deCampo DM, Becoats KT (2012) Revisiting the hippocampal-amygdala pathway in primates: association with immature-appearing neurons. Neuroscience 212:104–119. 10.1016/j.neuroscience.2012.03.040 22521814 PMC3367117

[B24] Fudge JL, Kunishio K, Walsh P, Richard C, Haber SN (2002) Amygdaloid projections to ventromedial striatal subterritories in the primate. Neuroscience 110:257–275. 10.1016/s0306-4522(01)00546-2 11958868

[B25] Fudge JL, Tucker T (2009) Amygdala projections to central amygdaloid nucleus subdivisions and transition zones in the primate. Neuroscience 159:819–841. 10.1016/j.neuroscience.2009.01.013 19272304 PMC2670361

[B26] Ghibaudi M, Marchetti N, Vergnano E, La Rosa C, Benedetti B, Couillard-Despres S, Farioli-Vecchioli S, Bonfanti L (2023) Age-related changes in layer II immature neurons of the murine piriform cortex. Front Cell Neurosci 17:1205173. 10.3389/fncel.2023.1205173 37576566 PMC10416627

[B27] Gómez-Climent MÁ, Castillo-Gómez E, Varea E, Guirado R, Blasco-Ibáñez JM, Crespo C, Martínez-Guijarro FJ, Nácher J (2008) A population of prenatally generated cells in the rat paleocortex maintains an immature neuronal phenotype into adulthood. Cereb Cortex 18:2229–2240. 10.1093/cercor/bhm255 18245040

[B28] Gouwens NW, et al. (2019) Classification of electrophysiological and morphological types in mouse visual cortex. Nat Neurosci 22:1182–1195. 10.1101/368456 31209381 PMC8078853

[B29] Gupta A, Proddutur A, Chang Y-J, Raturi V, Guevarra J, Shah Y, Elgammal FS, Santhakumar V (2020) Dendritic morphology and inhibitory regulation distinguish dentate semilunar granule cells from granule cells through distinct stages of postnatal development. Brain Struct Funct 225:2841–2855. 10.1007/s00429-020-02162-y 33124674 PMC7677165

[B30] Hattox AM, Nelson SB (2007) Layer V neurons in mouse cortex projecting to different targets have distinct physiological properties. J Neurophysiol 98:3330–3340. 10.1152/jn.00397.2007 17898147

[B31] Häusser M, Spruston N, Stuart GJ (2000) Diversity and dynamics of dendritic signaling. Science 290:739–744. 10.1126/science.290.5492.739 11052929

[B32] Izhikevich EM (2007) *Dynamical systems in neuroscience: the geometry of excitability and bursting*. Cambridge, MA: MIT Press.

[B33] Johnson DMG, Illig KR, Behan M, Haberly LB (2000) New features of connectivity in piriform cortex visualized by intracellular injection of pyramidal cells suggest that “primary” olfactory cortex functions like “association” cortex in other sensory systems. J Neurosci 20:6974–6982. 10.1523/JNEUROSCI.20-18-06974.2000 10995842 PMC6772836

[B34] Krahe R, Gabbiani F (2004) Burst firing in sensory systems. Nat Rev Neurosci 5:13–23. 10.1038/nrn1296 14661065

[B35] Latuske P, Toader O, Allen K (2015) Interspike intervals reveal functionally distinct cell populations in the medial entorhinal cortex. J Neurosci 35:10963–10976. 10.1523/JNEUROSCI.0276-15.2015 26245960 PMC6605276

[B36] Li WL, Chu MW, Wu A, Suzuki Y, Imayoshi I, Komiyama T (2018) Adult-born neurons facilitate olfactory bulb pattern separation during task engagement. Elife 7:e33006. 10.7554/eLife.33006 29533179 PMC5912906

[B37] Lisman JE (1997) Bursts as a unit of neural information: making unreliable synapses reliable. Trends Neurosci 20:38–43. 10.1016/S0166-2236(96)10070-9 9004418

[B38] Majak K, Moryś J (2007) Endopiriform nucleus connectivities: the implications for epileptogenesis and epilepsy. Folia Morphol 66:267–271. 18058746

[B39] Martí-Mengual U, Varea E, Crespo C, Blasco-Ibáñez JM, Nacher J (2013) Cells expressing markers of immature neurons in the amygdala of adult humans. Eur J Neurosci 37:10–22. 10.1111/ejn.12016 23066968

[B40] Miyamichi K, et al. (2011) Cortical representations of olfactory input by trans-synaptic tracing. Nature 472:191–196. 10.1038/nature09714 21179085 PMC3073090

[B41] Morey RA, Clarke EK, Haswell CC, Phillips RD, Clausen AN, Mufford MS, Saygin Z, Wagner HR, LaBar KS (2020) Amygdala nuclei volume and shape in military veterans with posttraumatic stress disorder. Biol Psychiatry Cogn Neurosci Neuroimaging 5:281–290. 10.1016/j.bpsc.2019.11.016 32029420 PMC8040290

[B42] Nacher J, Lanuza E, McEwen BS (2002) Distribution of PSA-NCAM expression in the amygdala of the adult rat. Neuroscience 113:479–484. 10.1016/S0306-4522(02)00219-1 12150768

[B43] Neville KR, Haberly LB (2004) Olfactory Cortex. In: *The synaptic organization of the brain* (Shepherd GM, ed), Ed 5, New York, NY: Oxford University Press.

[B44] Nowak LG, Azouz R, Sanchez-Vives MV, Gray CM, McCormick DA (2003) Electrophysiological classes of cat primary visual cortical neurons in vivo as revealed by quantitative analyses. J Neurophysiol 89:1541–1566. 10.1152/jn.00580.2002 12626627

[B45] Oh SW, et al. (2014) A mesoscale connectome of the mouse brain. Nature 508:207–214. 10.1038/nature13186 24695228 PMC5102064

[B46] Paré D, Gaudreau H (1996) Projection cells and interneurons of the lateral and basolateral amygdala: distinct firing patterns and differential relation to theta and delta rhythms in conscious cats. J Neurosci 16:3334–3350. 10.1523/JNEUROSCI.16-10-03334.1996 8627370 PMC6579143

[B47] Paré D, Pape HC, Dong J (1995) Bursting and oscillating neurons of the cat basolateral amygdaloid complex in vivo: electrophysiological properties and morphological features. J Neurophysiol 74:1179–1191. 10.1152/jn.1995.74.3.1179 7500142

[B48] Pitkänen A, Amaral DG (1998) Organization of the intrinsic connections of the monkey amygdaloid complex: projections originating in the lateral nucleus. J Comp Neurol 398:431–458. 10.1002/(SICI)1096-9861(19980831)398:3<431::AID-CNE9>3.0.CO;2-0 9714153

[B49] Piumatti M, Palazzo O, La Rosa C, Crociara P, Parolisi R, Luzzati F, Lévy F, Bonfanti L (2018) Non-newly generated, “immature” neurons in the sheep brain are not restricted to cerebral cortex. J Neurosci 38:826–842. 10.1523/JNEUROSCI.1781-17.2017 29217680 PMC6596233

[B50] Poirazi P, Mel BW (2001) Impact of active dendrites and structural plasticity on the memory capacity of neural tissue. Neuron 29:779–796. 10.1016/S0896-6273(01)00252-5 11301036

[B51] Price JL, Slotnick BM, Revial MF (1991) Olfactory projections to the hypothalamus. J Comp Neurol 306:447–461. 10.1002/cne.903060309 1713925

[B52] Rainnie DG, Asprodini EK, Shinnick-Gallagher P (1993) Intracellular recordings from morphologically identified neurons of the basolateral amygdala. J Neurophysiol 69:1350–1362. 10.1152/jn.1993.69.4.1350 8492168

[B53] Rotheneichner P, et al. (2018) Cellular plasticity in the adult murine piriform cortex: continuous maturation of dormant precursors into excitatory neurons. Cereb Cortex 28:2610–2621. 10.1093/cercor/bhy087 29688272 PMC5998952

[B54] Sakamoto M, Kageyama R, Imayoshi I (2014) The functional significance of newly born neurons integrated into olfactory bulb circuits. Front Neurosci 8:121. 10.3389/fnins.2014.00121 24904263 PMC4033306

[B55] Save L, Baude A, Cossart R (2019) Temporal embryonic origin critically determines cellular physiology in the dentate gyrus. Cereb Cortex 29:2639–2652. 10.1093/cercor/bhy132 29878074

[B56] Schaffer ES, Stettler DD, Kato D, Choi GB, Axel R, Abbott LF (2018) Odor perception on the two sides of the brain: consistency despite randomness. Neuron 98:736–742.e3. 10.1016/j.neuron.2018.04.004 29706585 PMC6026547

[B57] Sorrells SF, et al. (2019) Immature excitatory neurons develop during adolescence in the human amygdala. Nat Commun 10:2748. 10.1038/s41467-019-10765-1 31227709 PMC6588589

[B58] Sun YJ, Kim Y-J, Ibrahim LA, Tao HW, Zhang LI (2013) Synaptic mechanisms underlying functional dichotomy between intrinsic-bursting and regular-spiking neurons in auditory cortical layer 5. J Neurosci 33:5326–5339. 10.1523/JNEUROSCI.4810-12.2013 23516297 PMC3714095

[B59] Suzuki N, Bekkers JM (2006) Neural coding by two classes of principal cells in the mouse piriform cortex. J Neurosci 26:11938–11947. 10.1523/JNEUROSCI.3473-06.2006 17108168 PMC6674875

[B60] Suzuki N, Bekkers JM (2011) Two layers of synaptic processing by principal neurons in piriform cortex. J Neurosci 31:2156–2166. 10.1523/JNEUROSCI.5430-10.2011 21307252 PMC6633060

[B61] Swadlow HA, Gusev AG (2001) The impact of “bursting” thalamic impulses at a neocortical synapse. Nat Neurosci 4:1546–1726. 10.1038/86054 11276231

[B62] Tervo DGR, et al. (2016) A designer AAV variant permits efficient retrograde access to projection neurons. Neuron 92:372–382. 10.1016/j.neuron.2016.09.021 27720486 PMC5872824

[B63] Washburn MS, Moises HC (1992) Electrophysiological and morphological properties of rat basolateral amygdaloid neurons in vitro. J Neurosci 12:4066–4079. 10.1523/JNEUROSCI.12-10-04066.1992 1403101 PMC6575963

[B64] Zeldenrust F, Wadman WJ, Englitz B (2018) Neural coding with bursts—current state and future perspectives. Front Comput Neurosci 12:48. 10.3389/fncom.2018.00048 30034330 PMC6043860

[B65] Zeng H (2018) Mesoscale connectomics. Curr Opin Neurobiol 50:154–162. 10.1016/j.conb.2018.03.003 29579713 PMC6027632

[B66] Zhu X, et al. (2019) Rabies virus pseudotyped with CVS-N2C glycoprotein as a powerful tool for retrograde neuronal network tracing. Neurosci Bull 36:202–216. 10.1007/s12264-019-00423-3 31444652 PMC7056755

