# Supplementary figures and images for: Neuronal Subtypes and Connectivity of the Adult Mouse Paralaminar Amygdala
Source: eNeuro. 2024 Jun 12;11(6):ENEURO.0119-24.2024. doi: 10.1523/ENEURO.0119-24.2024 (PMC11208988; doi:10.1523/ENEURO.0119-24.2024)

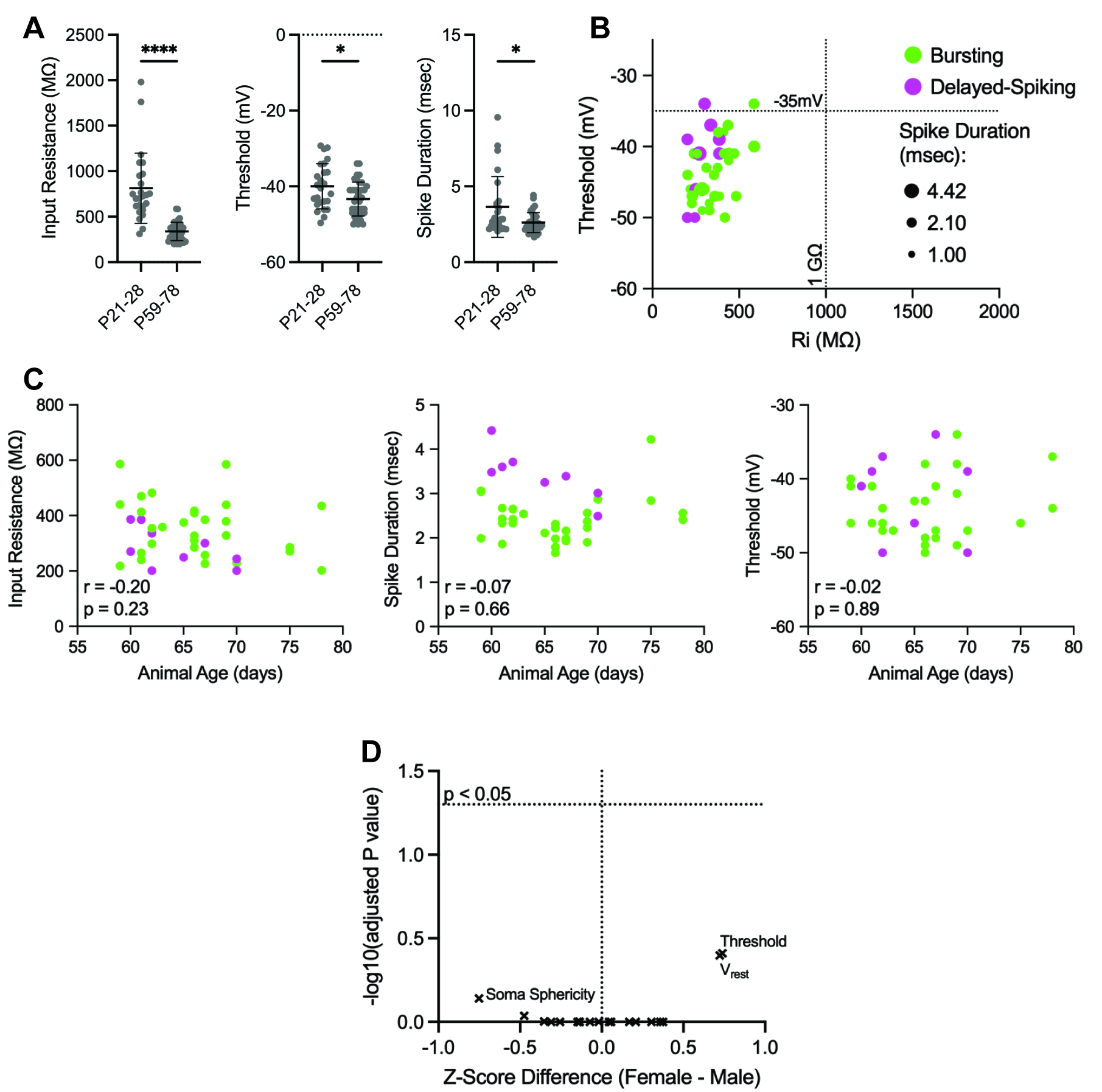

Supplement: Figure 1-1 — Maturational stages and sex differences in morpho-electric properties of adult (P59-78) mouse PL neurons. (A) T-tests comparing electrophysiological properties of adult PL neurons to the juvenile PL neurons in our previously published dataset (Alderman et al. 2024). (B) Threshold and spike duration plotted against input resistance. Dotted lines represent the cutoffs defining mature neurons in the juvenile PL from Alderman et al. (2024). (C) Input resistance, spike duration, and threshold plotted against animal age. (D) Volcano plot of q values (FDR-corrected p values, see methods) comparing sex differences in morphological and electrical properties of adult PL neurons. Horizontal dotted line represents an alpha cutoff of 0.05. Download Figure 1-1, TIF file. [file eneuro-11-ENEURO.0119-24.2024-s002.tif]

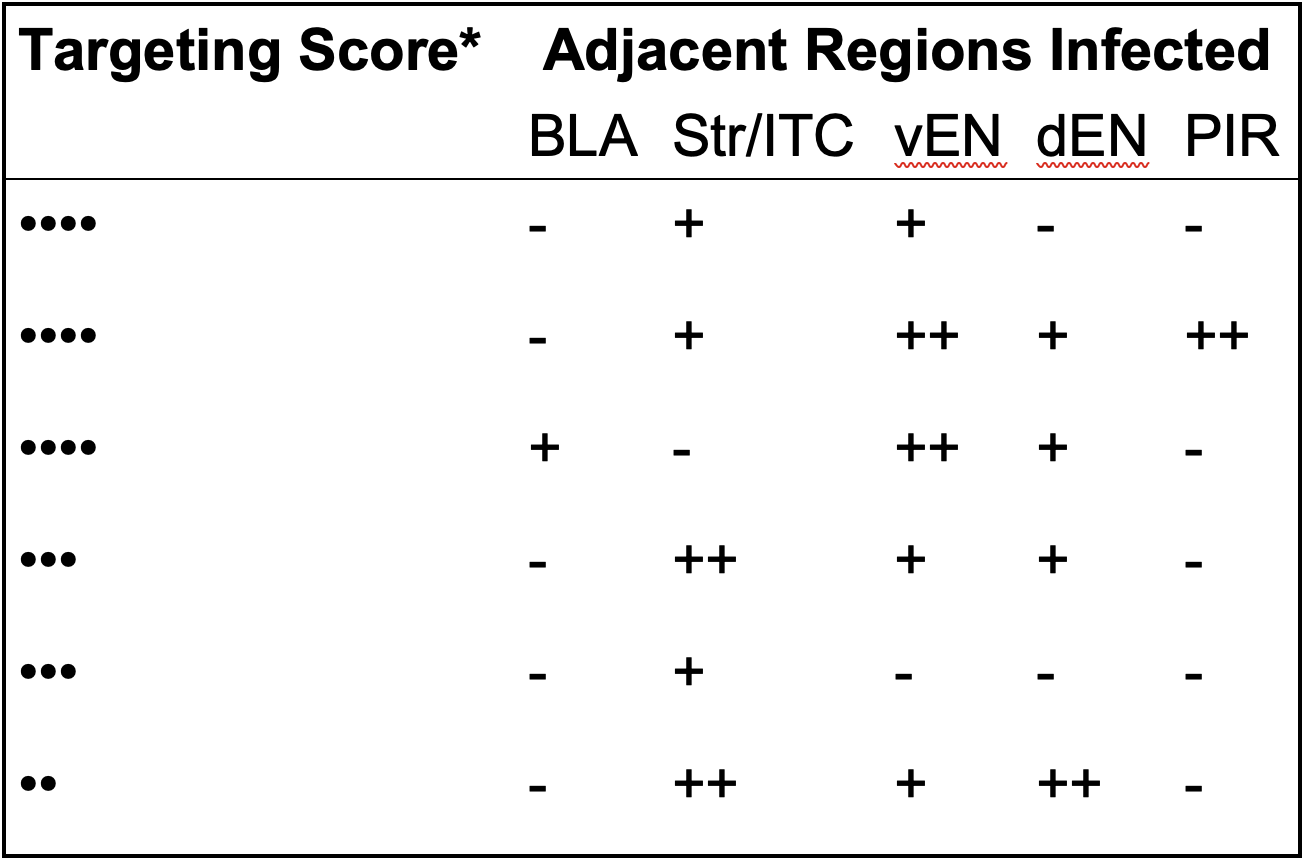

Supplement: Figure 3-1 — Injection targeting of retrograde virus to the adult mouse PL * Scoring criteria: •• 50% of PL targeted, ••• 75% of PL targeted, •••• > 90% of PL targeted. - no infected neurons, + 1-10 infected neurons, ++ 10-20 infected neurons (per 50 µm section) Download Figure 3-1, TIF file. [file eneuro-11-ENEURO.0119-24.2024-s003.tif]

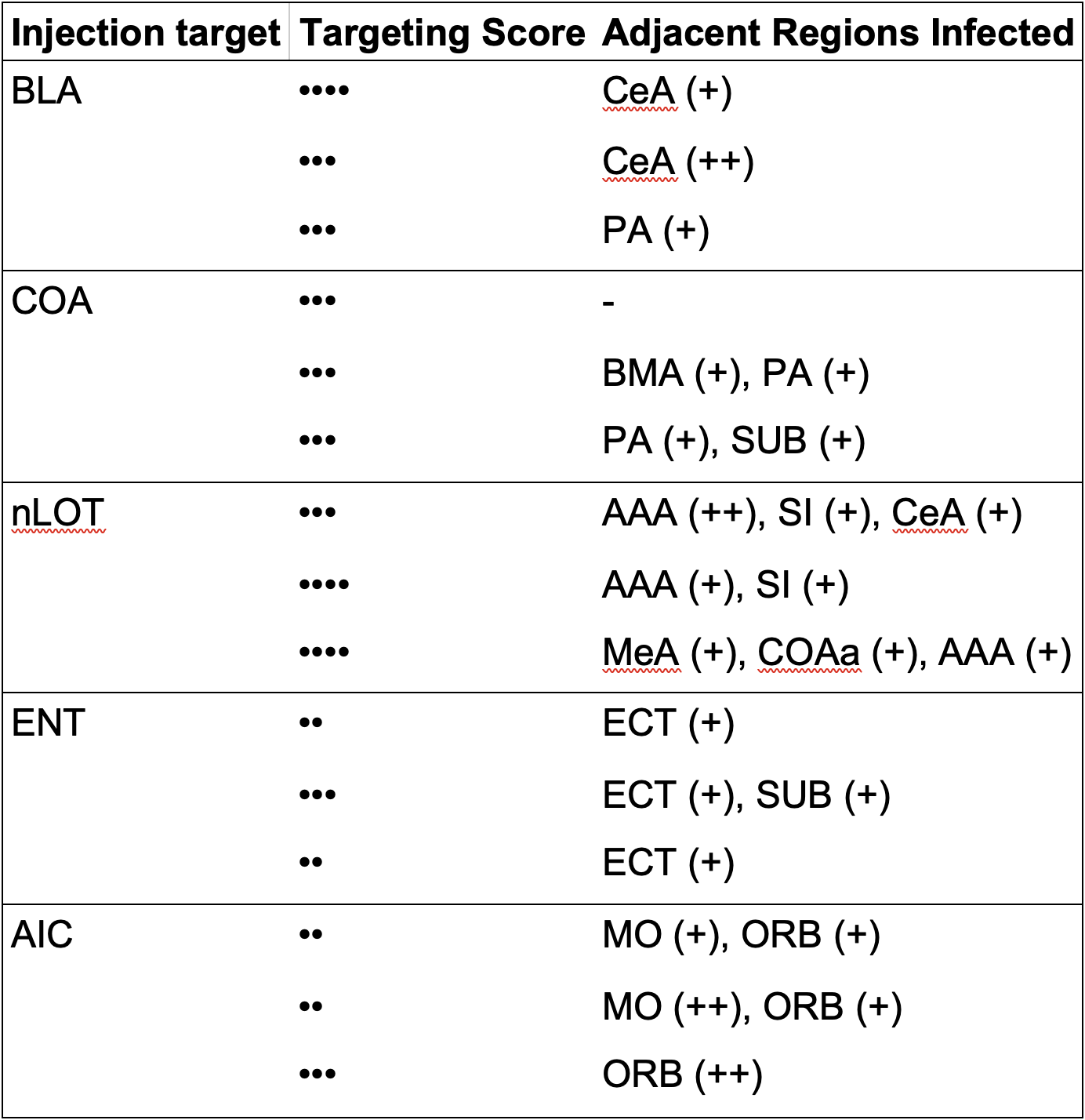

Supplement: Figure 4-1 — Injection targeting of anterograde virus to adult mouse PL input sources * Scoring criteria: •• 50% of region targeted, ••• 75% of region targeted, •••• > 90% of region targeted. - no infected neurons, + 1-10 infected neurons, ++ 10-20 infected neurons (per 50 µm section). Download Figure 4-1, TIF file. [file eneuro-11-ENEURO.0119-24.2024-s004.tif]

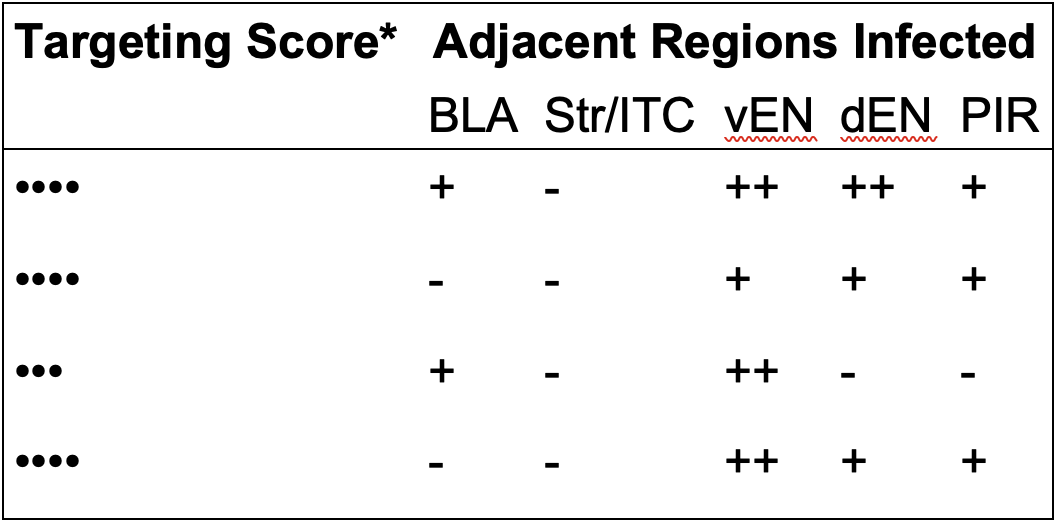

Supplement: Figure 5-1 — Injection targeting of anterograde virus to the adult mouse PL * Scoring criteria: • 25% of PL targeted, •• 50% of PL targeted, ••• 75% of PL targeted, •••• > 90% of PL targeted .- no infected neurons, + 1-10 infected neurons, ++ 10-20 infected neurons (per 50 µm section). Download Figure 5-1, TIF file. [file eneuro-11-ENEURO.0119-24.2024-s005.tif]
